# Relationships between Microbial Indicators and Pathogens in Recreational Water Settings

**DOI:** 10.3390/ijerph15122842

**Published:** 2018-12-13

**Authors:** Asja Korajkic, Brian R. McMinn, Valerie J. Harwood

**Affiliations:** 1 National Exposure Research Laboratory, Office of Research and Development, United States Environmental Protection Agency, 26 West Martin Luther King Drive, Cincinnati, OH 45268, USA; mcminn.brian@epa.gov; 2 Department of Integrative Biology, University of South Florida, 4202 East Fowler Ave, SCA 110, Tampa, FL 33620, USA; vharwood@usf.edu

**Keywords:** recreational water, fecal indicators, pathogens, relationships

## Abstract

Fecal pollution of recreational waters can cause scenic blight and pose a threat to public health, resulting in beach advisories and closures. Fecal indicator bacteria (total and fecal coliforms, *Escherichia coli*, and enterococci), and alternative indicators of fecal pollution (*Clostridium perfringens* and bacteriophages) are routinely used in the assessment of sanitary quality of recreational waters. However, fecal indicator bacteria (FIB), and alternative indicators are found in the gastrointestinal tract of humans, and many other animals and therefore are considered general indicators of fecal pollution. As such, there is room for improvement in terms of their use for informing risk assessment and remediation strategies. Microbial source tracking (MST) genetic markers are closely associated with animal hosts and are used to identify fecal pollution sources. In this review, we examine 73 papers generated over 40 years that reported the relationship between at least one indicator and one pathogen group or species. Nearly half of the reports did not include statistical analysis, while the remainder were almost equally split between those that observed statistically significant relationships and those that did not. Statistical significance was reported less frequently in marine and brackish waters compared to freshwater, and the number of statistically significant relationships was considerably higher in freshwater (*p* < 0.0001). Overall, significant relationships were more commonly reported between FIB and pathogenic bacteria or protozoa, compared to pathogenic viruses (*p*: 0.0022–0.0005), and this was more pronounced in freshwater compared to marine. Statistically significant relationships were typically noted following wet weather events and at sites known to be impacted by recent fecal pollution. Among the studies that reported frequency of detection, FIB were detected most consistently, followed by alternative indicators. MST markers and the three pathogen groups were detected least frequently. This trend was mirrored by reported concentrations for each group of organisms (FIB > alternative indicators > MST markers > pathogens). Thus, while FIB, alternative indicators, and MST markers continue to be suitable indicators of fecal pollution, their relationship with waterborne pathogens, particularly viruses, is tenuous at best and influenced by many different factors such as frequency of detection, variable shedding rates, differential fate and transport characteristics, as well as a broad range of site-specific factors such as the potential for the presence of a complex mixture of multiple sources of fecal contamination and pathogens.

## 1. Introduction

Approximately 39% of the United States (US) population and more than 50% of the global population live near a coastal area [1,2]. Coastal tourism accounts for 85% of all tourism revenue in the US [3], with the average beachgoer spending ~$35 per beach visit [4], resulting in a massive contribution to local economy and national gross domestic product [5,6]. During 2013, approximately 10% of US beach samples (out of total 116,230 samples collected) at 3485 beaches exceeded the US Environmental Protection Agency beach action value (BAV) for fecal indicator bacteria (FIB), indicating unacceptable water quality [5]. Similarly, a more recent report for the European Union (EU) indicated that ~15% of beach samples failed to meet the most stringent “excellent” quality standard at nearly 22,000 coastal beaches and inland sites across EU [7].

Because of a wide array of potential pathogens and typically low concentrations in environmental waters, direct monitoring of waterborne pathogens can be costly, technically challenging, and in some cases not feasible. Therefore, recreational waters are typically monitored for FIB levels instead. Monitoring is intended to ensure that the water body is safe for human recreational contact, and the resulting data are used to determine whether beach advisories or closures are needed. General FIB such as total coliforms, fecal coliforms, *Escherichia coli* and enterococci have been used worldwide for over a century in sanitary assessment of recreational waters [8,9,10,11,12]. The type of FIB measured and values used in recreational water guidelines vary by country [13]. Other general fecal microorganisms, such as *Clostridium perfringens* and various bacteriophages, are considered alternative indicator organisms, and are also frequently measured in various water quality monitoring programs worldwide [13,14,15,16,17,18,19]. However, FIB and alternative indicator organisms are common inhabitants of gastrointestinal tracts of mammals and birds [14,20], and their detection in environmental waters provides no information about the source of pollution. Considering that ambient waters can be influenced by multiple point and non-point pollution sources, identification of source is crucial for any remedial efforts and risk assessment determinations since not all fecal sources pose the same risk to human health. For example, human fecal pollution typically presents the greatest risk because of the possible presence of human viral pathogens, while cattle manure may be a close second because of the possible presence of zoonotic pathogens such as *Cryptosporidium* spp. and enteropathogenic *E. coli* [21]. Exposure to gull, chicken, and pig feces carries a known risk, because of possible presence of zoonotic pathogens associated with these animals including hepatitis E virus [22], *Campylobacter* spp., *Brucella* spp., pathogenic *E. coli* and *Salmonella* spp. [23,24,25]. Microbial source tracking (MST) has emerged in response to a need to identify the source(s) of fecal pollution to better safeguard human health and aid in remediation efforts. The majority of MST genetic markers target the 16S rRNA gene of *Bacteroides* spp., although some amplify other genes and, in some instances, viral targets [13,14]. Earlier technology centered on end-point PCR, which provides a binary, presence/absence result, but more recent studies estimate the concentration of a given MST genetic marker via real-time quantitative PCR (qPCR) [14]. Of note, more rapid technology in lieu of molecular enrichment followed by qPCR is also being developed for monitoring of *Staphylococcus aureus* and *Pseudomonas aeruginosa*, indicator species used to monitor the water quality in swimming pools [26].

The majority of waterborne disease outbreaks associated with recreational use of untreated waters (e.g., lakes and oceans) are caused by pathogenic microorganisms including bacteria, parasites, and viruses, while chemicals (including toxins) accounted for approximately 6% of outbreaks with confirmed etiology [27]. Among the pathogenic bacteria, virulent *Escherichia coli* serotypes (e.g., O157:H7), *Campylobacter* spp., *Legionella* spp., *Shigella* spp., *Salmonella* spp., and *Pseudomonas* spp. were most commonly identified etiologic agents [28,29,30,31,32]. While other protozoan species are occasionally identified as the cause (e.g., *Naegleria* spp.), [27,30], *Cryptosporidium* spp., followed by *Giardia* spp. are etiological agents for the majority of recreational waterborne outbreaks [28,29,31,32]. Regarding viral pathogens, noroviruses and adenoviruses were most frequently identified as causative agents in outbreaks where etiology was confirmed [27,30,32,33]. In treated waters (e.g., swimming pools and spas), *Cryptosporidium* spp. are most often identified as etiological agents [30,31,32], although noroviruses and adenoviruses are becoming more frequently detected [33]. It is important to note that etiological agents in nearly 30% of outbreaks in the US alone remain unidentified [27], and that sporadic recreational waterborne illnesses not associated with outbreaks are excluded from this report.

**Figure 1:**
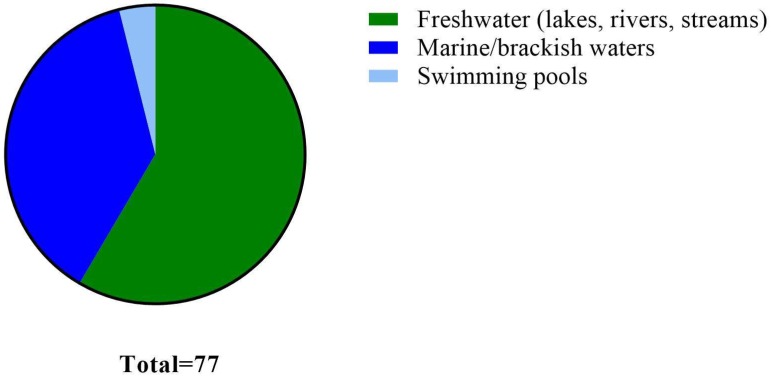
Documented relationships between various indicators and pathogens in freshwaters (*n* = 45), marine/brackish waters (*n* = 29) and swimming pools (*n* = 3).

Even though the concepts of FIB, alternative indicators, and MST markers were developed to indicate fecal contamination and its sources, the same paradigm is often employed to indicate pathogen presence under the assumption that indicators consistently covary with pathogen presence. The goals of this review are: (1) to examine reported relationships between various indicators and pathogen species to determine the feasibility of indicators as pathogen sentinels in recreational waters; and (2) to identify factors that affect this relationship (or lack thereof). In addition, we also queried epidemiological studies to determine which indicator(s) most commonly correlated with illness in recreational waters. Our search criteria mandated that each study measured at least one indicator (FIB or alternative) or MST marker along with at least one pathogen. We focused on studies conducted in waters intended for primary human contact (e.g., swimming, wading, diving, and surfing) such as beaches and swimming pools, but also included ambient waters used for secondary or non-contact (e.g., boating, fishing) human activities. Our methodology for collecting the manuscripts involved querying “PubMed” (www.ncbi.nlm.nih.gov/pubmed/) and “Google Scholar” (https://scholar.google.com/) databases for following keywords: “recreational water pathogens”, “recreational water viral pathogens”, “recreational water bacterial pathogens”, “recreational water protozoan pathogens”, “recreational water fungal pathogens”, “swimming pools pathogens”, “swimming pools viral pathogens”, “swimming pools bacterial pathogens, “swimming pools protozoan pathogens” and “swimming pools fungal pathogens” regardless of the year published. For the purposes of this review, a relationship is identified as a significant correlation (e.g., Pearson Product Momentum Correlation, Wilcoxon signed-rank tests) and/or significant predictive relationship (e.g., binary and other logistic regression modelling). Assumptions made in our analyses included the following: (1) all measurement strategies yielded equivalent results (e.g., various culture-based, molecular and microscopy were equally sensitive); and (2) data were not affected by characteristics of the water samples (e.g., we assumed that the water chemistry did not influence performance of the methods). In total, we collected 73 papers spanning over four decades of research from 25 countries: Argentina, Australia, Bolivia, Brazil, Canada, China, Cyprus, Democratic Republic of Congo, France, Greece, Germany, Hungary, Iceland, Italy, Japan, Luxembourg, Netherlands, New Zealand, Poland, Portugal, South Africa, Taiwan, United Kingdom, US, and Venezuela. The majority of studies were conducted in freshwaters (lakes, rivers and streams), followed by marine/brackish waters and swimming pools (Figure 1). Since some studies were conducted in both fresh and marine/brackish waters, they were included in each water type in Figure 1. This resulted in a total of 126 observations (i.e., report on a relationship between indicator and pathogen) since some studies were conducted in both, marine and freshwater, and/or measured more than one type of indicator or pathogen. The majority of observations (*n* = 52) did not report any relationship between indicator(s) and pathogen(s), while those that did, were split into relationships that were statistically significant (*n* = 30) and those that were not (*n* = 44). Statistically significant relationships (or the lack thereof) and rationale for the observed trends are further examined in the following sections.

## 2. Relationships between Indicators and Pathogens in Recreational Waters

We queried studies conducted in marine, brackish, freshwater, and swimming pool waters meeting our search criteria for FIB, alternative indicators, MST markers, and pathogen data. FIB levels were typically reported as colony forming units (CFU), or most probable number (MPN), depending whether studies measured concentrations using membrane filtration on selective-differential media or defined substrate technology (e.g., Enterolert and Colilert), respectively (Table 1 and Table 2). However, a few studies quantified general FIB using qPCR and expressed concentrations usually as gene copies per unit volume (Table 1 and Table 2). Alternative indicators of fecal pollution such as *C. perfringens* and different bacteriophages [34] were also of interest for inclusion to assess their reliability for estimating pathogen presence compared to general FIB. *C. perfringens* was measured using membrane filtration on selective-differential media with concentrations expressed as CFU per unit volume, while bacteriophage concentrations were usually measured via single or double agar layer (SAL, DAL) techniques, with data expressed as plaque forming units (PFU) per unit volume (Table 3).

Lastly, studies using molecular assays targeting MST markers were gathered to determine any potential relationships with pathogenic organisms. Depending on the detection assay used, MST data was reported as either presence/absence (end-point PCR) or as gene copies (qPCR) per unit volume. Assays for general MST markers included those targeting 16S rRNA gene of *Bacteroidales* spp. (i.e., Bac23F, GenBac3, AllBac), and pepper mild mottle viruses (PMMoV) (Table 4). For human-associated MST markers, most assays targeted 16S rRNA or other functional genes of *Bacteroidales* or *Bacteroidales*-like organisms (e.g., HF183, *gyrB*, *Bfra*, HF134, *B. thetaiotamicron*, BacHum-UCD, *B. dorei*, *B. uniformis*, *B. stericoris*, HumM2, HumM19), as well as 16S rRNA of human -associated *C. coccoides*, *nifH* gene of *Methanobrevibacter smithii*, and *esp* gene of *Enterococcus faecium* (Table 4). Bovine/ruminant-associated MST markers typically target 16S rRNA genes of *Bacteroidales* spp. (e.g., BacCow, CF128, and CF193) or toxin-genes of *E. coli* (e.g., LTIIa), while swine-associated MST markers target *Bacteroidales* spp. (e.g., PF163) or *E. coli* (e.g., STII) (Table 4). Since pets and waterfowl can influence water quality, dog-associated markers have been developed targeting 16S rRNA of *Bacteroidales* spp. (BacCan and DogBac), as well as seagull associated markers targeting 16S rRNA from *Catelicoccus marimammalium* or *Bacteroides* spp. (Gull) (Table 4).

We also gathered data for various bacterial, viral, and protozoan pathogens. For bacterial pathogens, we collected data on 10 genera (*Vibrio*, *Salmonella*, *Shigella*, *Mycobacteria*, *Pseudomonas*, *Escherichia*, *Aeromonas*, *Campylobacter*, *Legionella*, and *Listeria*). Measurement strategies ranged from culture-based (data reported as CFU, MPN or presence/absence) to end-point PCR (presence/absence) and qPCR (gene copies) (Table 1, Table 2, Table 3 and Table 4). For viral pathogens, we collected data on eight different species including enteroviruses, adenoviruses, noroviruses, hepatitis A and E, astroviruses, rotaviruses, reoviruses, and sapoviruses. Viral data were expressed as MPN (infectious viruses, ICC-[RT]-PCR), presence/absence (PCR) or gene copies (qPCR) (Table 1, Table 2, Table 3 and Table 4). The most frequently measured protozoan pathogens, *Cryptosporidium* spp. and *Giardia* spp. (oo)cysts, were usually enumerated using immunomagnetic separation, followed by staining, although in some instances, qPCR was also performed (Table 1, Table 2, Table 3 and Table 4). *Enterocytozoon bieneusi* was measured using similar detection methods to that of *Cryptosporidium* and *Giardia* (oo)cysts (Table 2), while two pathogenic amoeba species (*Acanthamoeba* spp. and *Naegleria fowleri*) were reported as presence/absence (i.e., PCR) (Table 1). Lastly, *Candida* spp. were enumerated using membrane filtration on selective-differential media and reported as CFU (Table 2). Sections below summarize our results regarding relationships that FIB, alternative indicators and MST markers have with pathogens and waterborne illness occurrence in freshwater, marine/brackish waters and swimming pools.

## 3. FIB and Pathogens in Freshwater

The relationships between FIB and various pathogens in freshwater, and the individual studies from which they were derived, are summarized in Table 1. Of the 41 studies, approximately one third (*n* = 18) [35,36,37,38,39,40,41,42,43,44,45,46,47,48,49,50,51,52] did not report any relationship between indicators and pathogens measured. Of the remaining 23 studies, thirteen reported positive relationship between at least one indicator and one pathogen [53,54,55,56,57,58,59,60,61,62,63,64,65], while ten did not find significant relationships [66,67,68,69,70,71,72,73,74,75]. Please see Table 1 (“relationship” and “comments” columns) for summary of relationships and other comments regarding studies that found no significant relationship or those that did not report it.

Studies that found significant, positive relationships most commonly reported it for *Cryptosporidium*/*Giardia* (oo)cysts and pathogenic *E. coli* spp., followed by *Salmonella* spp. and *Campylobacter* spp. (Table 1). Relationships were less frequently noted for *Shigella* spp. and adenoviruses, as well as non-fecal pathogens such as *Legionella* spp. and *Acanthamoeba* spp. (Table 1). *E. coli* was the FIB with the greatest number of significant relationships, followed by enterococci, fecal and total coliforms (Table 1). Significant relationships were reported between *E. coli* and pathogenic *E. coli* spp. (*n* = 7), *Cryptosporidium*/*Giardia* (oo)cysts (*n* = 6), *Salmonella* spp. (*n* = 6), *Campylobacter* spp. (*n* = 4), and adenoviruses (*n* = 2) (Table 1). Enterococci also had the greatest number of statistically significant relationships with pathogenic *E. coli* spp. (*n* = 5), *Cryptosporidium*/*Giardia* (oo)cysts (*n* = 4), *Salmonella* spp. (*n* = 3), *Campylobacter* spp. (*n* = 2), and adenoviruses (*n* = 1) (Table 1). Fecal coliforms correlated with pathogenic *E. coli* spp. (*n* = 5), followed by *Salmonella* spp. (*n* = 3), *Cryptosporidium*/*Giardia* (oo)cysts (*n* = 2), and *Campylobacter* and *Shigella* spp. (*n* = 1 each) (Table 1). Statistically significant relationships between total coliforms and different pathogens (pathogenic *E. coli* spp., *Salmonella* spp., *Campylobacter* spp., *Legionella*, and *Acanthamoeba* spp.) were reported only once for each pathogen (Table 1). The methodology employed did not appear to influence the outcome, as significant relationships were not more likely when both indicator and pathogen were measured by a similar technique (Table 1).

The frequency of significant relationships of FIB with bacterial or protozoan pathogens was similar; however, significant relationships with viral pathogens were less frequent (Fisher’s exact test, *p*: 0.0005–0.0022). While lack of relationship between FIB and pathogenic viruses is not surprising given the enormous differences between the two groups in terms of persistence in the environment and low levels of viral pathogens typically found in ambient waters, correlations between FIB and protozoan pathogens are more difficult to understand. Interestingly, when *Cryptosporidium* and *Giardia* spp. were detected, they were generally present in higher concentrations compared to viral pathogens. Significant relationships were reported more commonly for rivers and streams compared to lakes [58,59,60,62], but this trend was not statistically significant (Fisher’s exact test, two-tailed *p* = 0.085). Correlations appeared to be influenced by weather conditions, as most occurred during wet seasons and/or following rainfall events [53,61,63,67,75]. Not surprisingly, correlations were also more likely following sewage spills and/or wastewater discharges [54,56,57,61] and in waters impacted by agricultural operations [57,61], likely due to elevated FIB concentrations, greater likelihood of pathogen presence and the potential for location to be dominated by single fecal source.

## 4. FIB and Pathogens in Marine and Brackish Water

Table 2 lists general FIB and pathogen relationships reported in the datasets analyzed for marine and brackish waters. Of the 29 studies reviewed, almost half (*n* = 13) did not report statistical analysis of the relationships [52,76,77,78,79,80,81,82,83,84,85,86,87]. Within the remaining studies, ten did not find a relationship [64,70,88,89,90,91,92,93,94,95] while six reported a positive relationship between at least one indicator and one pathogen [59,96,97,98,99,100]. Statistical significance was reported less frequently in marine and brackish waters compared to freshwater (17 vs. 44) and the proportion of statistically significant relationships (compared to non-significant) was considerably higher in freshwater (Fisher’s exact test, *p* < 0.0001). Please see Table 2 (“relationship” and “comments” columns) for a summary of relationships and other comments regarding studies that found no significant relationship or those that did not report it.

Significant relationships with FIB were most commonly reported for *Salmonella* spp., followed by adenoviruses, and *Campylobacter* spp., *Vibrio* spp., *S. aureus*, and protozoan pathogens (Table 2). The most significant relationships with pathogens were reported for enterococci (*n* = 11), followed by *E. coli* (*n* = 4), and fecal coliforms (*n* = 2). No statistically significant relationships were reported for total coliforms. Significant relationships were reported between enterococci and adenoviruses (*n* = 8), *Salmonella* spp. (*n* = 4), *Cryptosporidium*/*Giardia* (oo)cysts (*n* = 4), *Campylobacter* spp. (*n* = 3), and *Candida* spp., *Vibrio* spp., *S. aureus*, noroviruses, and *E. bieneusi* (one observation each) (Table 2). *E. coli* formed significant relationships with *Salmonella* spp. and *Vibrio* spp. (*n* = 1 each) and adenoviruses (*n* = 2). Statistically significant relationships with fecal coliforms were reported only for adenoviruses (28.6%, *n* = 7). The methodology employed did not appear to influence the outcome; significant relationships were not more likely when both indicator and pathogen were measured by a similar technique (Table 2).

As expected, FIB had more significant relationships with bacterial pathogens compared to viral pathogens (Fisher’s exact, *p* = 0.0069), but there was no significant difference in other comparisons (i.e., FIB relationships with bacterial compared to protozoan pathogens, or FIB relationships with protozoan compared to viral pathogens). Of note, FIB most likely to correlate with pathogens were enterococci, which supports its recommended use to monitor marine recreational water quality. No clear trend for different marine water types (e.g., brackish waters and coastal beaches) was observed with respect to statistically significant indicator/pathogen relationships [59,96,98,99,100,101], suggesting that hydrological factors play less of a role compared to freshwaters. Similar to freshwater, the common trend among the studies reporting significant relationships was that they were conducted in waters impacted by fecal contamination [96,98,99], and when bather numbers were high [97], conditions likely to result in elevated FIB and pathogen levels.

## 5. Alternative Indicators and Pathogens in Marine, Brackish and Freshwater

Ten studies conducted in freshwater and fourteen studies conducted in brackish and marine waters measured at least one alternative indicator and one pathogen. In freshwater, four studies measured *C. perfringens*, five studies measured bacteriophage, and one study measured both (Table 3). In brackish/marine waters, the majority (*n* = 12) of studies measured coliphage (somatic, F-specific), followed by *C. perfringens* (*n* = 7), and phages infecting *Bacteroides thetaiotaomicron* (*n* = 1) (Table 3). Similar to FIB, more statistically significant relationships were reported in freshwater compared to brackish/marine waters (Fisher’s exact test, *p* = 0.0057). Please see Table 3 (“relationship” and “comments” columns) for summary of relationships and other comments regarding studies that found no significant relationship or those that did not report it.

In freshwater, half of studies (*n* = 5) did not report statistical analysis [41,45,47,49,102], four reported at least one statistically significant relationship [54,63,64,103], while a single study reported a non-significant relationship [39] (Table 3). Statistically significant relationships were reported more frequently for *C. perfringens*, followed by F-specific and somatic coliphages (Table 3). *C. perfringens* had positive relationships with *Campylobacter* spp. (*n* = 2), and *Listeria* spp., *Salmonella* spp. and pathogenic *E. coli* spp. (one observation each). F-specific coliphage correlated with noroviruses, *Cryptosporidium*/*Giardia* (oo)cysts, and *Campylobacter* spp. (one observation each) while somatic coliphage correlated only with adenoviruses (*n* = 2). Similar to FIB, the observed correlations occurred in waters affected by sewage discharge [54], following rainfall events [103], and were affected by season and sampling site [63].

In marine and brackish waters, approximately, half of studies (*n* = 6) did not report any statistical analysis [76,77,81,82,87,89], while three reported statistically significant relationships [79,90,100], and five reported non-significant relationships [64,88,91,92,94]. Two studies found significant relationships between F-specific coliphage and pathogens; one reported it with methicillin resistant *S. aureus* (MRSA), and *S. aureus* at a marine beach affected by fecal-impacted freshwater intrusion [79], while a second reported it for adenoviruses in water impacted by urban run-off [90] (Table 3). No studies noted a significant relationship between somatic coliphage and pathogens (Table 3). Only a single study conducted in Hawaii [100], a state that recommends using *C. perfringens* for monitoring ambient waters [104], found a relationship between this indicator, and two pathogens (*Campylobacter* spp., and *V. parahaemolyticus*) (Table 3). The methodology employed did not appear to influence the outcome in marine or freshwaters; significant relationships were not more likely when both indicator and pathogen were measured by a similar technique (Table 3). While there were insufficient data to perform statistical analyses regarding relationship of alternative indicators and different pathogen groups, F-specific coliphage tended to perform better compared to somatic coliphage and *C. perfringens*.

## 6. MST Markers and Pathogens in Marine, Brackish and Freshwater

The number of studies that measured MST marker(s) along with at least one pathogen is considerably smaller (*n* = 19; eight in freshwater, and 11 in brackish/marine/waters) compared to studies measuring FIB or alternative indicators (Table 4). The majority of MST measurements reported were for human-associated marker(s) (76.1%), followed by general MST markers (7.0%), cattle and dog associated MST markers (5.6%), and seagull and swine-associated MST markers (2.8%) (Table 4). Most frequently measured pathogens were viruses (adenovirus, enterovirus, noroviruses, hepatitis, and infectious enteric viruses) and bacteria (*E. coli*, *Campylobacter* spp., *Salmonella* spp., *V. vulnificus*, *S. aureus*, and *Legionella* spp.) with 22 measurements each, while *Cryptosporidium* and *Giardia* (oo)cysts were reported less frequently (*n* = 6) (Table 4). Irrespective of the water type, nine of these studies did not report statistical analyses for relationships between MST marker(s) and pathogens [51,76,77,79,80,83,86,88,105], and another seven reported non-significant relationship [48,56,57,70,72,98,99]. The remaining two studies reported statistically significant relationship [93,106] (Table 4). Please see Table 4 (“relationship” and “comments” columns) for summary of relationships and other comments regarding studies that found no significant relationship or those that did not report it.

Significant relationships between pathogens and human-associated MST markers were reported for HF183 and adenoviruses, at a marine beach impacted by non-point source(s) [93], and between HF183/HF134 and *Campylobacter* spp. in freshwater affected by livestock operations [106]. In the same freshwater study, cattle-associated MST markers (CF128, CF193) correlated with *E. coli* O157:H7, and *Salmonella* spp., while a general *Bacteroidales* MST marker (Bac32F) correlated with all three pathogens [106]. The methodology employed did not appear to influence the outcome; in other words, significant relationships were not more likely when both indicator and pathogen were measured by a similar technique (Table 4). There were insufficient data regarding relationship of MST markers and different pathogen groups (e.g., bacterial, viral and protozoan) to perform statistical analyses. While it may seem counter-intuitive that MST markers (particularly human-associated subset), were not generally correlated with pathogens, it is important to note that sensitivity and specificity of MST markers varies greatly [14]. Furthermore, many pathogens reported in these studies are zoonotic, making this relationship even more tenuous.

## 7. Various Indicators and Pathogens in Swimming Pools

Our search of literature for paired measurements of indicator(s) and pathogen(s) recorded for swimming pools yielded considerably fewer studies (*n* = 3), compared to ambient waters. None of the studies reported statistical analyses on relationships between indicators and pathogens. Two studies, both conducted in Italy, were performed on pools that were in compliance with microbiological requirements for *E. coli*, enterococci, *P. aeruginosa*, and *S. aureus* [107,108]. However, one study detected infectious *Simkania negevensis*, a bacterium related to *Chlamydia*, in nearly 43% of samples, while the second one measured Papillomaviruses in 64% of samples [108]. Interestingly, HPyVs were co-detected with Papillomaviruses in all the samples [108]. *L. pneumophila* and enteric viruses (adenovirus, norovirus, and enteroviruses) [108] were not detected. Examination of wading pools in Finland during a gastroenteritis outbreak detected Norovirus GII and astrovirus in ~83% and ~33% of samples, respectively [109]. *E. coli* was absent from samples collected ~2 weeks before the outbreak, but high concentrations (370–24,000 CFU/100 mL) were measured in two samples taken during the outbreak [109].

## 8. Relationship of Indicators with Illness

**Figure 2:**
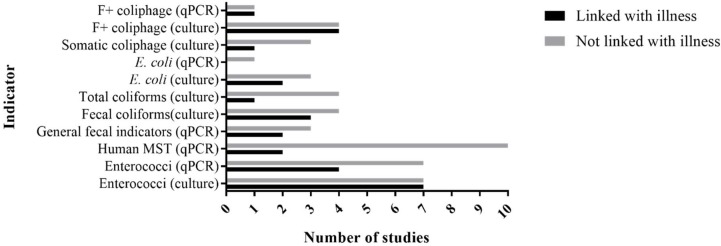
Summary of epidemiological studies reporting on linkage between illness and various indicator types.

To identify associations between the presence of general FIB, alternative indicators or MST markers with that of waterborne illness occurrence, various epidemiologic studies were collected from existing literature dating back to the early 1990s. For inclusion, it was required that the study measured at least one FIB, alternative indicator or MST marker (culture or molecular) in combination with an epidemiological survey of resulting illness from the recreational water exposure. In total, 17 studies [76,79,86,110,111,112,113,114,115,116,117,118,119,120,121,122,123,124] met these criteria and were included in analyses. One study each was conducted in Europe and Africa, and fifteen studies were conducted in the US. Since some of these studies were conducted in more than one water type, this resulted in the inclusion of 20 freshwater sites and 29 brackish/marine sites. Thirteen different microbiological assays were reported including those targeting: enterococci, fecal and total coliforms, *E. coli*, somatic and F+ coliphage, as well as various general and human-associated MST markers (Figure 2). In addition to gastrointestinal illnesses characterized by symptoms of diarrhea, vomiting, and stomach cramps, other waterborne illnesses included skin, ear and sinus infection [76,79,86,110,111,112,113,115,116,117,118,119,120,121,122,124]. For epidemiological studies, assays targeting enterococci were the most commonly recorded, with 25 instances of measurements of either culture based or molecular enterococci targets, followed by human-associated MST markers, F+ coliphage, fecal coliforms, general MST markers, total coliforms, culturable *E. coli*, somatic coliphage and finally *E. coli* qPCR signal (Figure 2).

Correlations between observed illness in these studies were most common with enterococci (10 studies out of 17) [79,86,110,111,113,114,116,117,120,121], followed by F+ coliphage (5 studies) [79,113,118,119,123] (Figure 2), suggesting that these two indicators may be better predictors of waterborne illness occurrence. Fecal coliforms, human-associated MST markers (Bsteri, BuniF2, and HF134), general MST marker (GenBac3), culturable *E. coli*, total coliforms, and somatic coliphage were correlated with illness less frequently (Figure 2). Twenty-seven indicator measurements across all studies were correlated with human illness, and 93% of these studies were conducted in waters with known point or non-point source contamination, contaminated surface/ground water flow or following wet weather events. Only six studies [79,86,117,118,119,124], all of which found relationship between indicator and illness, measured pathogens, in addition to recording illness information, and indicator organism concentrations. Only one of the six studies found a relationship between pathogens and illness or indicator concentrations. This is not surprising since, in these studies, pathogens were detected infrequently and at low concentrations. This illustrates the potential challenges of detecting relationships between indicators and pathogens in the field even when health relationships were observed with fecal indictors.

## 9. Factors that Influence Indicator and Pathogen Relationships

Most recreational waters at any given time are impacted by many different sources of fecal contamination (e.g., treated and untreated wastewater, agricultural operations, stormwater, and domestic and wildlife animals) and these influences can change depending on many different factors including precipitation, tidal flow and wind direction. In addition, each fecal source has its own set of indicators and the potential for different types of pathogens. Therefore, the more fecal sources a recreational water is impacted by, the more challenging it will be to show correlations between indicators and pathogens. Preceding sections described our findings regarding relationships between indicators and pathogens in recreational waters, as well as relationships between indicators and illness. Overall, FIB were better predictors of bacterial and protozoan pathogen presence (compared to viral), relationships were more probable under scenarios where both indicator and pathogen were likely to be present at higher concentrations, and enterococci and F-specific coliphage tended to be better predictors of waterborne illness occurrence compared to other indicators. The following sections examine various factors that are likely to influence the observed trends.

## 10. Detection Frequency and Concentrations of Indicators and Pathogens in Marine, Brackish and Freshwaters

The observed relationships between indicators and pathogens can be influenced by logistical factors that may confound determination of actual relationships, including study design and methodological limitations. Study design determines the frequency at which a target (FIB, alternative indicator, MST marker or pathogen) was measured (per study or cumulative multiple studies), while methodology employed influences likelihood of detection. We compiled studies that reported frequency of detection (or data that allowed calculation of frequency detection such as total number of samples and samples positive) for at least one FIB/alternative indicator/MST marker and at least one pathogen per sample(s), resulting in inclusion of 49 studies (Table 5). Microbial data collected were first grouped according to indicator (FIB, alternative or MST) or pathogen type (bacterial, viral or protozoan), and further organized according to the detection format employed (different types of culture-based or molecular). Table 5 describes the detection frequency (per study and per total cumulative samples) for microorganism targets (FIB, alternative indicators, MST, and pathogens) for both freshwater and brackish/marine waters.

Each FIB was detected at least once in 100% of studies, which was true for most of the microbial targets. General FIB were also the most frequently detected on a per sample basis, as they were found in 94.2% of samples across 13,823 measurements in marine and freshwaters (Table 5). In freshwater, detection frequency of FIB per sample was 95% across 11,920 measurements and it was somewhat lower in brackish/marine waters (93% across 2203 measurements) (Table 5). Detection frequency of alternative indicators per sample, irrespective of the water type, was considerably less than FIB, averaging 60.6% (across 2606 measurements); the difference between water types was also more pronounced than for FIB (freshwater detection frequency 83.7%/2366 measurements vs. marine 45.1%/240 samples) (Table 5). While the 2705 samples analyzed for MST markers in marine and freshwaters were similar to alternative indicators, the overall detection frequency average (42.9%) was considerably lower (Table 5). The frequency of detection and total number of samples collected in each water type (37.4%/1355 in freshwater vs. 47.3%/1350 in marine water) was similar (Table 5).

Irrespective of the water type, bacterial pathogens were measured more often (9280 total samples), compared to viral (3462) and protozoan (3400) pathogens, although the frequency of detection across different pathogen groups was similar (33.8%, bacterial; 29.6%, viral; and 28.9%, protozoan (Table 5)). There also appeared to be no appreciable difference in detection frequency between the water types for any of the pathogen groups, although considerably more samples were collected in freshwater (Table 5). For bacterial pathogens, frequency of detection across 8936 total samples collected in freshwater was 35.2%, compared to 30.3% in 344 marine/brackish water samples (Table 5). Similarly, viral pathogens were detected in 33.1% freshwater samples (out of 2952) and 23.6% of 510 marine water samples (Table 5). Lastly, protozoan pathogens were detected in 34.6% of 3,134 freshwater samples and 24.4% of 266 marine water samples (Table 5).

**Figure 3:**
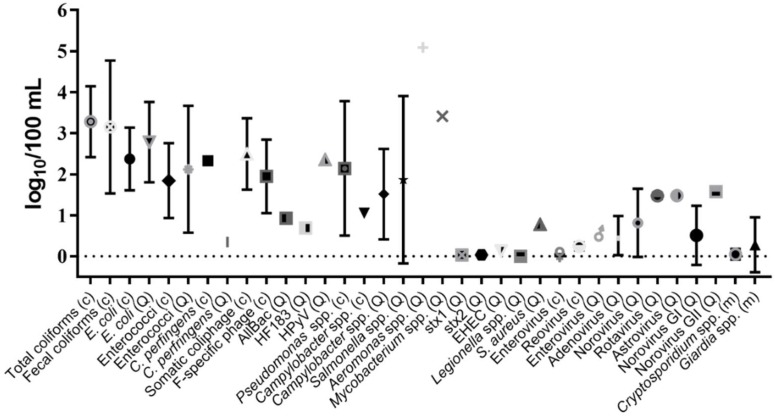
Mean concentration of FIB, alternative indicators, MST markers, bacterial, viral and protozoan pathogens in freshwater. Error bars represent standard deviation (c, culture-based; Q, qPCR; m, microscopy).

**Figure 4:**
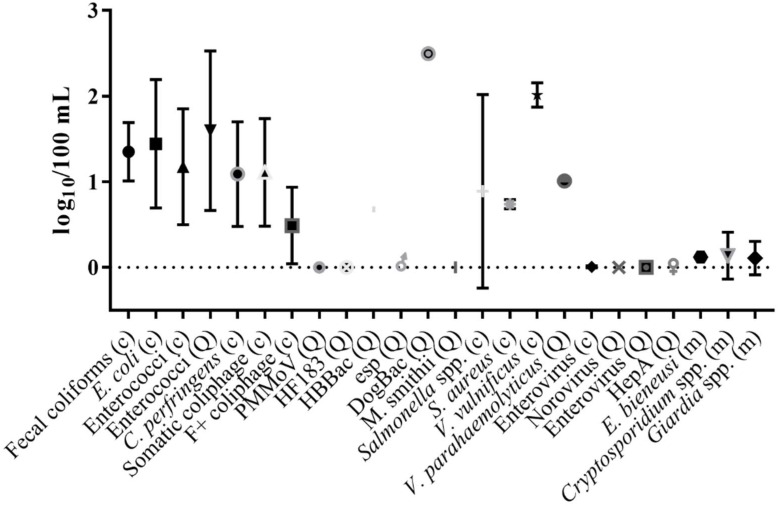
Mean concentration of FIB, alternative indicators, MST markers, bacterial, viral and protozoan pathogens in marine and brackish waters. Error bars represent standard deviation (c, culture-based; Q, qPCR; m, microscopy).

A subset of studies examined (*n* = 33) reported concentration data in the body of the manuscript, tables or supplemental materials, allowing graphs to be created displaying average densities per organism and water type (Figure 3 and Figure 4). Concentrations of indicators were on average 1–3 log_10_ higher than pathogen concentrations for both water types. Both indicators and pathogens in marine waters were found at slightly lower levels (0.5–1 log_10_) than those observed in freshwater (Figure 3 and Figure 4 and Table 6). Within an indicator group, concentrations of FIB ranged from not detected (ND (observed only for enterococci)) to 5.39 log_10_ per 100 mL (Table 6), and total coliform levels were the highest, followed by fecal coliforms, *E. coli*, and enterococci (Figure 3 and Figure 4). Alternative indicator concentrations were lower than FIB (ranging from ND–3.29 log_10_ per 100 mL (Table 6)), and *C. perfringens* levels were higher than somatic and F-specific coliphage (Figure 3 and Figure 4). MST marker concentrations were reported less frequently and were more variable, ranging from ND–2.50 log_10_ copies per 100 mL (Table 6 and Figure 3 and Figure 4). Bacterial pathogen concentrations (range: ND–5.09 log_10_ per 100 mL) were higher than viral (range: ND–1.58 log_10_ per 100 mL) and protozoan pathogens (range: ND–1.93 log_10_ per 100 mL), in both marine and freshwater (Table 6, Figure 3 and Figure 4).

As evidenced by the examples above, readily detected microorganisms are more likely to be measured and frequency of detection of a given microorganism is influenced by the concentration and distribution of the target in the sample types tested, as well as the limit of detection of the method used. Culture methods such as membrane filtration can have a low limit of detection, e.g., 1 CFU/100 mL, and can reliably detect FIB in water samples with minimal contamination. Conversely, pathogens are generally present sporadically and in lower levels than fecal indicators. These types of targets require high-throughput filtration methods that can achieve large concentration factors, with the tradeoff that limits of detection are generally quite high. In addition, the volumes sampled, and the concentration strategy used can vary between studies and can affect the sensitivity of a given method. These logistical factors frequently result in unbalanced comparisons in which the indicator organism is frequently detected, but the pathogen is not. Therefore, the disconnect between indicators and pathogens may not be due to a true lack of relationship in many cases, but to methodology that is much more suited to detecting indicators than pathogens.

## 11. Microbial Levels in Fecal Material

The observed relationships between indicators and pathogens can also be affected by factors intrinsic to the organisms themselves, including levels in various hosts, as well as shedding frequencies and duration. FIB are commensal inhabitants of the GI tract of humans and other animals and as such are shed continually in feces. Levels of fecal coliforms, *E. coli*, and enterococci typically found in human feces range 10^5^–10^9^ CFU per gram [13], while levels detected in untreated wastewater are somewhat lower (10^5^–10^8^ CFU per 100 mL) [13,128]. The concentration of FIB in animal excreta is lower still, ranging from 10^4^ to 10^7^ CFU per gram, depending on the animal host [13]. Alternative indicators are also commensal organisms of the GI tract but are typically found in lower concentrations and are more influenced by diets and physiologies of the host [13,14,128]. For example, *C. perfringens* levels in animal and human feces range from undetectable to 10^8^ CFU per gram [13], while coliphages were absent from some animal feces and primary wastewater effluents [128,129] and typically did not exceed ~10^3^ PFU/mL of untreated wastewater [128]. MST markers target different fecal microorganisms that are strongly associated with particular hosts [14] and the human-associated subset is reported to range from 10^3^ to 10^10^ gene copies per gram of feces or 100 mL of untreated wastewater, while animal-associated MST markers range from 10^4^–10^9^ gene copies per gram of feces depending on the sensitivity of individual markers and geographic region [13].

Pathogens may cause symptomatic or asymptomatic infection of their human and animal hosts. Shedding rates can vary widely, although levels found in the wastewater are typically several orders of magnitude lower than any indicator species [128,130,131,132,133] likely due to the sporadic nature of pathogen occurrence and detection compared to indicators. Additionally, only a small part of the population is infected with pathogens at any given time, resulting in considerable variation in the levels of pathogens, particularly when originating from relatively small populations. Differential shedding of pathogens from infected hosts is also contributing to the occurrence of pathogens in recreational waters. For example, shedding rates for human viral pathogens can be as high as 10^11^ viral particles per gram of feces in the case of adenoviruses [134], while shedding rates of bacterial pathogens are typically lower [133], as cattle excreting >10^4^ CFU per gram of feces *E. coli* O157:H7 are considered to be “super-shedders” [135]. *Cryptosporidium* and *Giardia* (oo)cyst shedding rates by the infected individuals can range from 10^6^ to 10^11^ per gram of feces [132] and are typically higher in animal hosts compared to human [131,136], although not all (oo)cysts excreted by animals are zoonotic [131,137].

Shedding duration of viruses can vary from weeks to months [133], and some viruses display distinct seasonal trends (e.g., infectious enteroviruses are more prevalent in wastewater in summer and early fall) [134]. Similar to pathogenic viruses, excretion of (oo)cysts is typically long term [132]. Shedding duration of bacterial pathogens is shorter with median values typically reported to be ~2 weeks, although in some instances it can last considerably longer [138,139]. Similar to viral and protozoan pathogens, shedding is affected by many different factors including diet and age of the host [140,141], temperature [140], as well as composition of gut microbiome [142]. Infectious dose of different pathogens is also variable and typically the lowest for viruses [134,143], medium range for protozoan pathogens and generally highest for bacterial pathogens [143], although *E. coli* O157:H7, with a low infectious dose, is an exception [144]. The infectious dose of viral, bacterial and protozoan pathogens is dependent on many factors, including individual strains and health status of the host [134,143].

## 12. Susceptibility to Environmental Stressors

While wastewater treatment processes generally result in some removal of indicators and pathogens [128,145], sanitary sewer and combined sewer overflows, along with other infrastructure failures can result in release of indicators and pathogens into ambient waters. In addition, different indicator and pathogen groups exhibit variable susceptibilities to disinfection strategies. Bacteria are generally susceptible to chlorination and UV treatment [146]. Protozoa and viruses are typically most susceptible to UV treatment [146,147], with the notable exception of adenoviruses [148]. Once indicators and pathogens are released into ambient waters, a new panoply of biotic and abiotic environmental factors affects fate and transport characteristics, including ambient sunlight, indigenous microbiota (i.e., predation and competition interactions), temperature, salinity, nutrient levels, location (water column vs. sediment), source of fecal pollution and resilience of individual organisms.

Ambient sunlight and associated UV radiation typically act to increase the decay rates, although the magnitude of this effect is influenced by the environmental conditions [149] and measurement strategies [150,151]. For example, viable cells and culturable/infectious organisms typically display the effects of UV damage more readily than their corresponding nucleic acids. Interactions with indigenous microbiota also increase decay rates, although this was predominantly shown for FIB, MST markers and some bacterial pathogens, (e.g., [152,153,154,155]) with inconclusive data for other organisms (e.g., various bacteriophages and *C. parvum* [156,157]). Influx of nutrients (in the form of organic carbon, nitrogen and phosphorus) can result in extended persistence [158,159,160], and potentially mitigate the effects of biotic interactions [161] but this assertion was not tested in detail for organisms other than culturable FIB. Temperature and location affect decay rates of most organisms tested (e.g., FIB, bacteriophage, viral pathogens, MST markers) almost unilaterally with greater persistence at lower temperatures [150,162,163,164,165,166] and in the sediments and sands compared to the water column (recently reviewed in [167]).

Similar to the effect of ambient sunlight, salinity (and the associated ionic content of brackish and marine waters) affected the decay rates of culturable/infectious FIB, alternative indicators, MST markers and pathogens more so than their corresponding nucleic acids [168,169,170,171,172,173,174]. The effect of source of fecal pollution has been studied on FIB and MST markers, and indicators originating from ruminants are more persistent compared to those from other fecal sources (e.g., dog, seagull, and human) [169,175,176,177,178,179], although different human sources (e.g., feces, septage, and sewage) elicit different decay rates [152]; analogous information for alternative indicators and pathogens is still missing. Finally, studies that compared decay of various indicators to pathogens directly under the same experimental conditions are rare and report conflicting results. For instance, in one study, *E. coli* O157:H7 persisted longer than FIB (e.g., *E. coli* and enterococci) in freshwater [180], but another group reported similar trend in the freshwater sediments but not the water column [181]. Another group reported no difference in decay between FIB, various MST markers and *C. jejuni*, *S. enterica* and adenovirus in freshwater [151]. Others reported considerably faster decay of *C. jejuni* (but not *C. coli* or *Salmonella* spp.) than FIB and MST markers, irrespective of the water type [171]. As exemplified above, variable responses of different indicator and pathogen groups to these stressors and the resulting differential decay rates further confound the indicator paradigm.

## 13. Conclusions

FIB and alternative indicator organisms (*C. perfringens* and coliphages) have been used for over a century, and continue to be used today as indicators of general fecal pollution in many applications, including the assessment of sanitary quality of recreational waters [8]. MST markers are used to identify source(s) of fecal pollution and are a more recent addition to the monitoring toolbox available to water quality managers and other practitioners in the field [14]. The goal of this review is to two-fold. Our primary objective was to examine reported relationships between various indicators and pathogens in recreational waters to determine the value of different indicators as surrogates for pathogen presence. Secondly, we aimed to more closely inspect different factors that may have an impact on this relationship.

The majority of the studies either did not report a relationship, or they reported a statistically non-significant relationship. Among the studies that observed statistically significant relationships, it was considerably more common in freshwater compared to marine waters. General FIB tended to form statistically significant relationships more commonly with bacterial and protozoan pathogens, (compared to viral pathogens) and this difference was statistically significant. Alternative indicators and MST markers correlated with pathogens less frequently, although it occurred more in freshwater than marine/brackish waters. Overall, statistically significant relationships were detected more frequently in waters known to be impacted by fecal pollution and following wet weather events, both scenarios under which indicators and pathogens are more likely to be co-detected.

Among factors influencing these relationships frequency of detection and variable concentrations of indicators and pathogens were identified as major contributing factor. Not surprisingly, general FIB were measured and detected more frequently than any other indicator or pathogen (generally in >90% of samples) and were also reported at higher concentrations, irrespective of the water type. Alternative indicators were also frequently detected in samples (>70%), while MST markers were measured and detected less frequently, and in lower concentrations than FIB or alternative markers (frequently in <10% of samples). Pathogen detection frequencies were similarly low. Low frequency of detection affects the ability to establish relationships between the frequently-detected and infrequently-detected analytes, as the dataset becomes left-censored (biased toward non-detects values). What looks like “absence” is frequently an artifact of comparing an analyte with high density (e.g., FIB) with one of low density (e.g., pathogen). Better concentration and recovery methods for the infrequently-detected analytes may provide a more realistic picture of the relationships among these various microorganisms in environmental waters. Finally, concentrations in feces and wastewater, shedding rates and patterns of various indicator and pathogen groups differ, as do their fate and transport characteristics in secondary habitats. Indicators are typically present in higher concentrations than any of the pathogen groups, and are also shed constantly, or more frequently, compared to pathogens. Upon entry into the secondary habitats, a host of biotic and abiotic factors differentially affects persistence of indicators and pathogens, further confounding the indicator–pathogen paradigm. Lastly, another important factor impacting the ability to establish relationships between indicators and pathogens is the realization that most locations are impacted by multiple sources of fecal contamination. Although it is difficult to measure the impact of multiple fecal inputs, tools such as sanitary surveys and GIS mapping have the ability to indicate potential point and non-point sources of fecal pollution and future MST studies should improve our understanding of the impacts of multiple fecal sources.

To further our understanding of indicator and pathogen relationships, future studies measuring these microorganisms in recreational waters should evaluate and report the existence (or lack thereof) of such relationships. Other considerations include careful selection of targeted pathogens and methodology used to quantify them. Furthermore, providing the data on a per sample basis (rather than descriptive statistics of a dataset) in at least supplementary materials, will enable metanalyses, which may yield a more robust estimate of a true state of indicator/pathogen paradigm. Lastly, while standardized and sensitive methods exist for FIB detection and enumeration in recreational waters, analogous procedures for alternative indicators, MST markers and pathogens are still missing. Standardization of detection and quantification methods suitable for each indicator/pathogen group can enable more accurate evaluation of any statistically significant relationships between these two groups.

## Figures and Tables

**Table 1:** Relationships between fecal indicator bacteria and various pathogens in freshwater.

Indicator(s) ^1^	Pathogen(s) ^1^	Location	Relationship ^2^	Comments	Reference
*E. coli* ^a^	*V. cholerae*^b^, *Salmonella* spp. ^b^, *Shigella* spp. ^b^	Apies River and tributaries, South Africa	NR	All three pathogens frequently co-detected. Concentrations of *E. coli* and pathogens higher during wet season.	[35]
*E. coli* ^a^	*Cryptosporidium* and *Giardia* (oo)cysts ^c^	Sure River and tributaries, Luxembourg	*Cryptosporidium* and *Giardia* oo(cysts) correlated with each other and with *E. coli*.	*Cryptosporidium* and *Giardia* oo(cysts) frequently co-detected. Concentrations highest during the wet season.	[53]
*E. coli* ^d^	*M. avium*^d^, *P. aeruginosa*^d^, *Salmonella* spp. ^d^	“Aohai See” Lake, China	No significant correlation.	All three pathogens frequently co-detected. Different seasonal patterns observed for *E. coli* and all three pathogens.	[66]
*E. coli* ^e^	*Campylobacter* spp. ^a^, *Giardia* and *Cryptosporidium* (oo)cysts ^c^	Avon River, New Zealand	*E. coli* strongly correlated with the all three pathogens.	All three pathogens frequently detected in samples where *E. coli* > 550 CFU. Higher concentrations of all pathogens found in samples during a large sewage discharge due to earthquake.	[54]
*E. coli* ^a^	*Giardia* and *Cryptosporidium* (oo)cysts ^c^	Canals connecting Rapipat and Rangsit canals, Thailand	NR	Highest levels of FIB and pathogens found in the most populated area.	[36]
*E. coli*^e,d^, enterococci ^e,d^	*Cryptosporidium* and *Giardia* (oo)cysts ^c^	Chicago area waterways system, various rivers and lakes, USA states	Stronger correlations between FIB and *Giardia* spp. than *Cryptosporidium* spp.	Correlations generally stronger in samples not impacted by the wastewater effluent. Associations between pathogens and enterococci generally stronger than with *E. coli*	[55]
Fecal coliforms ^e^, *E. coli* ^e^ and enterococci ^e^	Pathogenic *E. coli* ^b^	St Joseph River and Galien River watersheds in Michigan and Indiana, USA	No significant correlation.	Two or more virulence genes frequently co-detected. Samples with lower FIB levels, typically had a lower proportion of virulence genes.	[67]
*E. coli*^e^, enterococci ^e^	Pathogenic *E. coli* ^b^, *Cryptosporidium* and *Giardia* (oo)cysts ^c^	Various streams in Pennsylvania, USA	FIB correlated with all pathogens.	Samples exceeding recreational water quality guidelines more likely to contain pathogenic *E. coli* genes but not *Cryptosporidium* and *Giardia* (oo)cysts. Affected by non-point source and run-off following snow melt and rain events as well.	[56]
*E. coli* ^d^	*S. enterica*^d^, *Aeromonas* spp. ^d^, *M. avium* ^d^, *P. aeruginosa* ^d^	Ao, Hong and Tao lakes in Beijing, China	No significant correlation.	*E. coli* and *Aeromonas* spp. co-detected in all samples. Higher concentrations of *E. coli* and pathogens in particle attached fraction of the sample.	[68]
Total coliforms ^a^, *E. coli*^a^, enterococci ^a^	*P. aeruginosa* ^a^	Sauce Grande lagoon, Argentina	NR	*Pseudomonas aeruginosa* was co-detected with all FIB in all samples. All FIB positively correlated with temperature.	[37]
Total coliforms ^a^, fecal coliforms ^a^	Human enteroviruses ^b^, adenoviruses ^b^	Altamaha River, USA	No significant correlation.	Viruses co-detected in 26% of samples. Presence of viruses directly related to dissolved oxygen and streamflow, but inversely related to temperature, rainfall in the last 30 days.	[69]
Total coliforms ^a^, fecal coliforms ^a^, *E. coli* ^a^, enterococci ^e^	*Salmonella* spp. ^b^, *S. aureus* ^b^	Msunduzi River, South Africa	NR	Presence of *Salmonella* spp., *Staphylococcus aureus* and enterococci frequently coincided with fecal coliform and *E. coli* levels above 1000 MPN/100 mL. *Salmonella* spp. not detected in drier, colder months when fecal coliform and *E. coli* levels were below 1000 MPN/100 mL.	[38]
Fecal coliforms ^e^, *E. coli*^e^, enterococci ^e^	Pathogenic *E. coli* ^b^	Various rivers in Georgia, Kansas, Michigan, North Carolina, New Jersey, Ohio, South Dakota, Tennessee, Texas and Virginia, USA	Only eaeA gene positively correlated with FIB.	Multiple pathogenic genes co-detected in samples meeting and exceeding FIB guidelines. Some pathogenic genes also detected in three samples that met FIB guidelines. All sites known to be impacted by human fecal pollution and agricultural operations (upstream).	[57]
Enterococci ^e^	*Campylobacter* spp. ^d^	Various ponds, rivers and creeks in Florida, USA	No significant correlation.	Enterococci and *Campylobacter* spp. frequently co-detected, but enterococci were also detected in samples negative for *Campylobacter*. Florida DOH guidelines would not indicate *Campylobacter* spp. presence.	[70]
Fecal coliforms ^e^, enterococci ^e^	Infectious enteroviruses ^a^, total enteroviruses ^d^, hepatitis A ^d^, Norwalk I and II ^d^, astroviruses ^d^, rotaviruses ^d^	Rivers in France	NR	Infectious enteroviruses were not detected in samples with elevated fecal coliforms concentrations. Fecal coliforms, but not enterococci fluctuated seasonally.	[39]
Total coliforms ^e^	*Legionella* spp. ^d^	Hot spring recreational facilities, Taiwan	NR	Most samples failed to meet Taiwan CDC guidelines of 0 total coliforms per 100 mL. No *Legionella* spp. detected in two samples that met Taiwan CDC coliform guidelines.	[40]
*E. coli* ^a^	Pathogenic *E. coli* ^d^, *Shigella* spp. ^d^, *Salmonella* spp. ^d^, *C. jejuni* ^d^, *L. pneumophila* ^d^, *L. monocytogenes* ^d^, *V. cholerae* ^d^, and *V. parahaemolyticus* ^d^	Various streams around Lake Miyajimanuma, Japan	No significant correlation.	Various pathogen genes frequently co-detected. Temporal variation in pathogen concentration was observed with higher levels detected in colder months and when geese were present (not significant).	[71]
*E. coli* ^d^	Human adenovirus ^d^, *Giardia* and *Cryptosporidium* (oo)cysts^c^	Rivers in France	NR	Highest concentration of adenovirus found at two urban sites. Generally higher concentration of *Giardia* compared to *Cryptosporidium*.	[41]
Total coliforms ^e^	*Acanthamoeba* spp. ^b,d^, *Naegleria* spp. ^b,d^, *Legionella* spp. ^b,d^	Puzih River and two hot springs recreational facilities, Taiwan	*Acanthamoeba* spp. and *Legionella* spp. significantly associated with total coliforms in hot spring samples but not river. No significant correlation for *Naegleria* spp.	*Legionella* detection was significantly correlated with water temperature and more likely in the presence of *Vermamoeba vermiformis*.	[58]
*E. coli*^a^, enterococci ^a^	Human adenoviruses ^b,d^, noroviruses GII ^b^, and enteroviruses ^b^	Danube, Berettyo, Koros and Tisza Rivers and two rivulets (Koloska and Keki), Hungary	NR	In ~1/3 of samples at least two viral targets were co-detected. ~42%, 12% and 14% of designated recreational waters contained adenoviruses, enteroviruses and noroviruses.	[42]
*E. coli*^a^, enterococci ^e^	*Salmonella* spp. ^b^	Transitional and inland waters, Portugal	Significant correlation commonly observed in waters classified as “poor” and “sufficient” but also seen in waters classified as “good” or “excellent”	Higher geometric mean of FIB in *Salmonella* spp. positive samples than in *Salmonella* spp. negative samples. Even though significant correlation was reported, *Salmonella* was also detected in water samples with “good” and “excellent” water quality.	[59]
Total coliforms ^e^, *E. coli* ^e^	Pathogenic *E. coli* ^b^	Sauce Chico River, El Belisario Stream and San Bernardo Stream, Argentina	NR	Shiga-toxin producing *E. coli* found in samples where *E. coli* counts generally exceeded or were close to the US EPA recommended limits. High *E. coli* counts correlated with rainy season.	[43]
Total coliforms ^a^, fecal coliforms ^e^	Rotavirus ^b^, human Adenovirus ^b^, human Astrovirus ^b^, Norovirus ^b^	Rivers and streams, Brazil	No significant correlation.	Rotaviruses detected most frequently, followed by adenoviruses. The majority of samples exceeded recommendations for recreational waters from the standard methods used for the examination of water and wastewater of 5000 and 1000 MPN per 100 mL for total and fecal coliforms.	[73]
*E. coli*^e^, enterococci ^e^	*Pseudomonas* spp. ^e^	Lake Ma Vallee, Democratic Republic of Congo	NR	Samples met European Directive 2006/7/CE for *E. coli* and enterococci. *Pseudomonas* spp. detected at only one site which coincidentally had the highest *E. coli* and enterococci concentrations.	[44]
*E. coli* ^a^	Pathogenic *E. coli* ^d^, *C. jejuni* ^d^, *Shigella* spp. ^d^, *Salmonella* spp. ^d^	Michigan, Superior, Huron and Erie lakes, USA	Beach seasonal mean *C, jejuni* abundance correlated with beach seasonal *E. coli* concentration and at one beach pathogenic *E. coli* abundance was positively correlated with daily *E. coli* concentrations	High degree of beach specific temporal variability in pathogenic gene concentrations.	[60]
Fecal coliforms ^a^	Pathogenic *E. coli* ^b^, *Shigella* spp. ^b^ and *Salmonella* spp. ^b^	La Paz River basin, Bolivia	The occurrence of pathogenic bacteria associated with fecal coliform densities.	Approximately 50% of pathogenic bacteria resistant to at least two antibiotics. Pathogens were frequently detected during rainy season at sites impacted by anthropogenic activities.	[61]
*E. coli*^a^, enterococci ^a^, fecal coliforms ^a^	*Cryptosporidium* spp. ^c^, *Salmonella* spp. ^b^, *Campylobacter* spp. ^a^	Lake Parramatta, Australia	NR	Only *Salmonella* spp., detected sporadically in waters with relatively low FIB concentrations. FIB concentration higher during wet weather.	[45]
*E. coli* ^e^	*Campylobacter* spp. ^d^, *Legionella* spp. ^d^, adenovirus ^d^, *Cryptosporidium* spp. ^d^	River, lake ponds and a wadi in Netherlands	NR	*Campylobacter* spp. detected in all samples, *Cryptosporidium* spp. never detected, other pathogens detected sporadically.	[46]
Fecal coliforms ^e^, *E. coli*^e^, enterococci ^e^	*Campylobacter* spp. ^b^, *Salmonella* spp. ^b^, *E. coli* O157:H7 ^d^, *Cryptosporidium* and *Giardia* (oo)cysts ^c^, astroviruses ^b^, hepatitis A ^b^ and E ^b^ viruses, rotavirus ^b^, norovirus ^b^, enterovirus ^b^	Canals and lakes, Netherlands	NR	Arboviruses, hepatitis A and E viruses and *E. coli* O157:H7 were not detected in any of the samples. Rotavirus, norovirus, enterovirus *Salmonella* and *Campylobacter* spp. detected sporadically. Infectious enteroviruses found in one sample. Low concentration of *Cryptosporidium* and *Giardia* (oo)cysts detected in samples that complied with the European bathing water legislation.	[47]
Total coliforms ^e^	*L. pneumophila* ^d^	Puzih River and hot spring recreational areas, Taiwan	Total coliforms and *L. pneumophila* significantly correlated.	*L. pneumophila* detected in > 90% of samples. *L. pneumophila* and total coliforms also correlated with turbidity.	[62]
*E. coli*^e^, enterococci ^e^	Shiga toxin genes ^d^	Lake Erie and tributaries, USA	No significant correlation.	Abundance and distributions of shiga-toxin genes highly variable. The majority of samples positive for shiga toxin genes were below the advisory threshold levels for *E. coli* and enterococci.	[74]
*E. coli*^e^, enterococci ^e^, fecal coliforms ^e^	*Cryptosporidium* and *Giardia* (oo)cysts ^c^, infectious enteroviruses ^a^, *Salmonella* spp ^a^	Lake Carroll, Tampa, FL	NR	Higher concentrations of indicators and more frequent pathogen detection following rain events.	[48]
*E. coli* ^e^	Human adenovirus ^d^, human enterovirus ^d^, Norovirus GI and GII ^d^	Delaware Lake, Madison Lake and East Fork Lake, USA	No significant correlation.	Adenoviruses detected more frequently than enteroviruses, followed by noroviruses. Human adenovirus and enterovirus correlated.	[72]
*E. coli*^a^, total coliforms ^a^, enterococci ^e^	*Campylobacter* spp. ^a^, *Salmonella* spp. ^a^, *P. aeruginosa* ^a^, *Cryptosporidium* and *Giardia* (oo)cysts ^c^, *Aeromonas* spp. ^e^	River Ruhr and barrier lakes, Germany	NR	All sampling sites achieved “sufficient” bathing water quality for enterococci but not *E. coli*. With the exception of *Aeromonas* spp., detection of all other pathogens was sporadic. Precipitation preceding sampling event resulted in elevated concentration of total coliforms, *E. coli*, enterococci, *Aeromonas* spp. and *Cryptosporidium* and *Giardia* (oo)cysts.	[49]
*E. coli*^e^, enterococci ^e^	*Pseudomonas* spp. ^e^, Norovirus ^d^	Geothermal pools, Iceland	NR	High concentrations of *Pseudomonas* spp. detected in samples that also contained high FIB counts. Norovirus was not detected.	[50]
*E. coli*^d^, enterococci ^d^	*S. aureus*^d^, *Salmonella* spp. ^d^, noroviruses ^d^	Prickett Creek, USA	NR	No correlation between *E. coli* and enterococci.	[51]
Total coliforms ^e^, fecal coliforms ^e^, *E. coli* ^e^, enterococci ^e^	*L. monocytogenes*^b^, *Salmonella* spp. ^b^, *E. coli* O157:H7 ^b^, *Campylobacter* spp. ^b^, *Cryptosporidium* and *Giardia* (oo)cysts ^c^	South Nation River basin, Canada	Weak relationships, but mostly positive (except *L. monocytogenes*).	The fraction of samples that contained an indicator when pathogen was detected was highest for the protozoan parasites. Relationships dependent on season and site.	[63]
*E. coli*^a^, enterococci ^a^	Human adenoviruses ^b^	Various rivers and lakes in France, Germany, Italy, Netherlands, Poland, United Kingdom	Concentrations of all indicators correlated with frequency of adenovirus detection.	> 50% of samples positive for adenovirus.*E. coli* concentrations higher than enterococci.	[64]
*E. coli*^a^, enterococci ^a^	Adenoviruses ^d,f^, norovirus GI ^b^ and GII ^b^	Various rivers and lakes in France, Germany, Italy, Netherlands, Poland, United Kingdom	NR	Both viruses frequently detected in samples that met “good” water quality guidelines for both *E. coli* and enterococci. Adenoviruses detected more frequently than noroviruses.	[52]
Total coliforms ^a^, fecal coliforms ^a^, *E. coli* ^a^, enterococci ^e^	*Salmonella* spp. ^f^, pathogenic *E. coli* ^f^, *Cryptosporidium* and *Giardia* (oo)cysts ^c^	Wanzhou watershed, China	Significant but weak correlations between indicators and *Salmonella* spp. and pathogenic *E. coli*.	*Cryptosporidium* and *Giardia* (oo)cysts detected in samples with low indicator concentrations. Concentrations of indicators influenced by rainfall.	[65]
Total coliforms ^a^	*Cryptosporidium* and *Giardia* (oo)cysts ^c^	Lake Tianjin, China	No significant correlation	Significant correlation between *Cryptosporidium* and *Giardia* (oocysts). *Giardia* detected more frequently.	[75]

^1^ Data reporting: most probable number (MPN) ^a^, Presence/absence ^b^, total (oo)cysts ^c^, gene copies ^d^, colony forming units (CFU) ^e^, Integrated cell culture (ICC)/MPN PCR ^f^. ^2^ NR (not reported).

**Table 2:** Relationships between fecal indicator bacteria and various pathogens in brackish and marine waters.

Indicator(s) ^1^	Pathogen(s) ^1^	Location	Relationship ^2^	Comments	Reference
Fecal coliforms ^e^, *E. coli* ^e^, enterococci ^a,d,e^	*V. vulnificus*^b^, *S. aureus*^e^, enterovirus ^d^, norovirus ^d^, hepatitis A virus ^d^, *Cryptosporidium* and *Giardia* (oo)cysts ^c^	Virginia Key Beach, Florida, USA	NR	When HPyV, *V. vulnificus*, and *Giardia* spp. were detected so were all indicators and alternative indicators. When FIB levels exceeded regulatory standards, HPyVs and pathogens also detected.	[77]
Fecal coliforms ^e^, *E. coli* ^e^, enterococci ^d,e^	*V. vulnificus*^b^, *S. aureus*^e^, enterovirus ^d^, norovirus ^d^, hepatitis A virus ^d^, *Cryptosporidium* and *Giardia* (oo)cysts ^c^	Coastal Beaches, Miami Dade County, Florida, USA	NR	When enterococci levels by qPCR and CS exceeded MDL, *Cryptospordium*, Giardia, enteroviruses and *V. vulnificus* were co-detected.	[76]
Enterococci ^e^	*Cryptosporidium* and *Giardia* (oo)cysts ^c^	Coastal beaches, Venezuela	No significant correlation.	Presence of *Cryptosporidium* and *Giardia* were significantly correlated.	[88]
*E. coli ^e^*, enterococci ^e^	*C. albicans*^b^, *Salmonella* spp. ^b^	Saronicos Gulf, Athens, Greece	Enterococci but not *E. coli* correlated with *Salmonella* spp., but not *C. albicans*.	Pathogens detected in waters of “good” and “excellent” quality.	[96]
Total coliforms ^a^, fecal coliforms ^a^	*Salmonella* spp. ^b^	Canals around Galveston Bay, TX, USA	NR	*Salmonella* spp. detection occurred (nearly 100%) when FC concentrations >2000/100 mL.	[78]
Enterococci ^a^	*C. parvum*^c^, *G. lamblia*^c^, *G. duodenalis*^c^*E. bieneusi*^c^	Maryland, US Chesapeake Bay, USA	*C. parvum*, *G. duodenalis* and *E. bieneusi* correlated with enterococci counts.	Correlations observed especially apparent with high bather numbers in water.	[97]
Enterococci ^a,d^, *E. coli* ^a,d^, fecal coliforms ^a^, total coliforms ^a^	*L. pneumophila*^b^, *S. aureus*^b^, MRSA ^b^, adenovirus ^b^, enterovirus ^d^, Hepatitis A ^d^, Norovirus ^d^	Malibu beach, California USA	NR	No indicator used had a significant correlation with GI illness in swimmers or any reference pathogen.	[79]
Enterococci ^e^	*Campylobacter* spp. ^d^,	Florida, Quietwater Beach, USA	No significant correlation.	Enterococci co-detected with pathogenic *Campylobacter* spp., but levels of enterococci were not indicative of levels of *Campylobacter* present.	[70]
*E. coli*^a^, enterococci ^a^	*Salmonella* spp. ^b^, *Campylobacter* spp. ^a^, *Cryptosporidium* and *Giardia* (oo)cysts ^c^, adenoviruses ^a^ enteroviruses ^a^	Estuaries, Melbourne Australia	No significant correlation.	Changes in FIB concentrations associated with changes in temperature, flow, humidity and rainfall.	[89]
Total coliforms ^e^, fecal coliforms ^e^, *E. coli* ^e^, enterococci ^e^	Adenoviruses ^a^	Southern California coastal waters, USA	No significant correlation.	5 of 12 sites, FIB exceeded CA recreational water quality limits.	[90]
Total coliforms ^e^, fecal coliforms ^e^, enterococci ^e^	Adenoviruses ^b^, enteroviruses ^b^, hepatitis A ^b^	Rivers and creeks in California, USA	No significant correlation.	FIB and viral pathogen detection associated with storm events. Total and fecal coliforms correlated with each other but not enterococci.	[91]
Total coliforms ^a^, fecal coliforms ^a^, enterococci ^a^	Enterovirus ^b^, adenoviruses ^b^	Newport Bay, California, USA	No significant correlation	FIB concentrations showed strong seasonal pattern, associated with winter storms Total and fecal coliforms correlated with each other but not enterococci.	[92]
Fecal coliforms ^e^, *E. coli* ^e^, enterococci ^e^	*Salmonella* spp. ^b^, enteric viruses ^b^	Ben T. Davis and Bahia beaches, Florida, USA	NR	*Salmonella* spp. not detected. Coxsackie B4 detected following major sewage spill FIB correlated with rainfall.	[80]
Fecal coliforms ^e^, enterococci ^e^	*Cryptosporidium* and *Giardia* (oo)cysts ^c^ enteroviruses ^a^	Sarasota Bay, Florida, USA	NR	FIB co-detected with all samples positive for enteric pathogens.	[81]
*E. coli*^e^, enterococci ^e^	*Salmonella* spp. ^e^	Coastal Waters, Portugal	Levels of FIB correlated with presence of *Salmonella* spp., especially in waters deemed “poor” or “sufficient” compared to “excellent”.	*Salmonella* spp. also detected in samples classified as “Good” or “Excellent”.	[59]
Fecal coliforms ^e^ *E. coli* ^e^, enterococci ^e^	Adenovirus ^b^	Hillsborough River and St. Johns River, Florida, USA	Presence of adenovirus strongly correlated with concentrations of all three FIB.	Samples collected in waters with known human fecal pollution, all FIB exceeded regulatory standards.	[98]
*E. coli*^e^, fecal coliforms ^e^, enterococci ^e^	Adenovirus ^b^	Hillsborough River, FL Tampa Bay Beach, Florida, USA	*E. coli*, enterococci and fecal coliforms correlated with adenovirus.	All FIB concentrations exceeded regulatory standards in samples.	[99]
Enterococci ^e^, fecal coliforms ^e^, total coliforms ^e^, *E. coli* ^e^	Adenovirus ^b^	Avalon and Doheny Beaches, California	No significant correlation.	FIB concentrations frequently exceeded recreational water quality guidelines. Adenoviruses frequently detected at Doheny beach, but not Avalon.	[93]
Total coliforms ^e^, fecal coliforms ^e^, enterococci ^e^, *E. coli* ^e^	Enteroviruses ^e^	Coastal beaches, Barcelona, Spain	NR	All samples with elevated FIB levels also had high levels of somatic and F-specific phage present. 55% of samples having infectious virus beach quality was within EU standards for levels of FIB.	[82]
Enterococci ^e^, fecal coliforms ^a^, *E. coli* ^a^, total coliforms ^a^	Enterovirus ^a^, *Cryptosporidium* and *Giardia* (oo)cysts ^c^	St. Lucie Estuary, Florida, USA	No significant correlation.	Viruses detected in samples where FIB levels were within regulatory limits.	[94]
Enterococci ^a,d^	Adenovirus ^b^, Norovirus ^d^, *Cryptosporidium* and *Giardia* (oo)cysts ^c^, *C. jejuni, Salmonella* spp. ^b^, *S. aureus* ^b^, *E. coli* 0157-H7 ^b^	Coastal beaches, Florida, USA	NR	FIB and pathogens co-detected Seawater samples taken near sewage discharges.	[83]
Enterococci ^e^	*V. vulnificus*^e^, *V. parahaemolyticus*^e^	Chesapeake, Bay, MD, USA	NR	Enterococci were co-detected with *V. vulnificus* and *V. parahaemolyticus*.	[84]
Enterococci ^e^	*V. vulnificus*^e^, *V. parahaemolyticus*^e^	Chesapeake, Bay MD, USA	NR	Enterococci were co-detected with *V. vulnificus* and *V. parahaemolyticus*. All *V. vulnificus* isolates susceptible to 14 of 26 antibiotics and *V. parahaemolyticus* to 11 of 26 antibiotics. All samples positive for enterococci and *Vibrio* spp. and within local recreational water quality guidelines.	[85]
Total coliforms ^e^, fecal coliforms ^e^, *E. coli* ^e^, enterococci ^e^	Hepatitis A ^b^, Norovirus GI ^b^	Coastal beaches, Lisbon, Portugal	No significant correlation.	All samples considered “good” quality based on local recreational water quality guidelines.	[95]
Enterococci ^a,e^	*S. aureus* ^e^	Coastal beaches, Miami, Florida, USA	NR	*S. aureus* was found in 37% of total samples, 1.1% positive for MRSA. Enterococci had a positive correlation with reports of skin illness.	[86]
*E.coli*^e^, enterococci ^e^	*Salmonella* spp. ^a^, *Campylobacter* spp. ^a^, *S. aureus* ^e^, *V. vulnificus* ^e^, *V. parahaemolyticus* ^e^	Hawaii streams, USA	*Salmonella, Campylobacter* and *C. jejuni* positively associated with enterococci and marginally associated with *S. aureus*. *V. vulnificus* was positively associated with all FIB, *V. parahaemolyticus* with *E. coli*.	Detection of at least one pathogens occurred in 21 of 22 streams tested.	[100]
Fecal coliforms ^e^, enterococci ^e^	Enteroviruses ^b^	Florida Keys, Florida, USA	NR	Enterovirus co-detected with fecal coliforms, enterococci. No sites in violation of water quality standards.	[87]
*E. coli*^a^, enterococci ^a^	Human Adenovirus ^b^	Coastal beaches in Cyprus, Italy, Portugal, Spain and United Kingdom	No significant correlation.	FIB levels significantly lower in seawater than in freshwater samples.	[64]
*E. coli*^e^, enterococci ^e^	Human adenovirus ^b^ norovirus ^b^ GI and GII	Coastal beaches in Cyprus, Italy, Portugal, Spain and United Kingdom	NR	Beaches considered “clean” based on FIB levels were positive for both adenovirus and noroviruses. Freshwater sites had higher frequency of virus detection than marine sites.	[52]

^1^ Data reporting: most probable number (MPN) ^a^, Presence/absence ^b^, total (oo)cysts ^c^, gene copies ^d^, colony forming units or plaque forming units (CFU/PFU) ^e^, Integrated cell culture (ICC)/MPN PCR ^f^. ^2^ NR (not reported).

**Table 3:** Relationship of alternative indicators of fecal pollution and pathogens in freshwater and marine/brackish waters.

Indicator(s) ^1^	Pathogen(s) ^1^	Location	Relationship ^2^	Comments	Reference
**Freshwater**					
*C. perfringens*^e^, F-RNA coliphage ^e^	*Campylobacter* spp. ^a^, *Giardia* and *Cryptosporidium* (oo)cysts ^c^	Avon River, Christchurch, New Zealand	F-RNA more strongly correlated with all three pathogens than *C. perfringens*.	F-RNA concentrations typically higher than *C. perfringens*. Study conducted in river affected by sewage discharge.	[54]
Somatic coliphage ^e^	Infectious enteroviruses ^a^, total enteroviruses ^d^, hepatitis A^d^, Norwalk I and II ^d^, astroviruses ^d^, rotaviruses ^d^	Rivers in France	No significant correlation.	Enterovirus genomes and somatic coliphage frequently co-detected. Infectious enteroviruses and hepatitis A, Norwalk I and II, astroviruses, rotaviruses detected in only one or two samples.	[39]
*C. perfringens* ^e^	Human adenovirus ^d^, *Giardia* and *Cryptosporidium* (oo)cysts ^c^	Rivers in France	NR	Highest concentration of protozoan parasites and *C. perfringens* found at the site with high proportion of agricultural operations, forests and semi-natural environments.	[41]
Somatic and F+ coliphage ^e^	Noroviruses ^f^, rotaviruses ^f^, infectious reoviruses and enteroviruses ^a^	Maas and Waal Rivers, Netherlands	NR	Both coliphages and all viruses co-detected in all samples. Coliphage concentrations higher than pathogenic virus concentrations.	[102]
Somatic and F+ coliphage ^e^	Noroviruses ^d^, adenoviruses ^d^, astroviruses ^d^, rotaviruses ^d^	Marine Reservoir and tributaries, Singapore	F+ coliphage positively correlated with norovirus concentrations.	Higher statistical correlation observed between enteric viruses than between enteric viruses and coliphages. Noroviruses most abundant, followed by rotaviruses. Wet weather concentration of coliphage and viruses higher than dry weather concentration, but difference is not statistically significant.	[103]
*C. perfringens* ^e^	*Cryptosporidium* spp. ^c^, *Salmonella* spp. ^b^, *Campylobacter* spp. ^a^	Lake Parramatta, Australia	NR	No *Cryptosporidium* detected which coincided with the low *C. perfringens* concentrations. *C. perfringens* concentration lower than FIB.	[45]
Somatic and F+ coliphages ^e^	*Campylobacter* spp. ^a^, *Salmonella* spp. ^b^, *E. coli* O157:H7 ^d^, *Cryptosporidium* and *Giardia* (oo)cysts ^c^, astroviruses ^b^, hepatitis A and E viruses ^b^, rotavirus ^b^, norovirus ^b^, enterovirus ^a^	Canals and lakes, Netherlands	NR	Somatic coliphage detected more frequently and at higher concentrations compared to F+ coliphage. Highest concentrations of bacteriophages occurred following a heavy rainfall.	[47]
*C. perfringens* ^e^	*Campylobacter* spp. ^a^, *Salmonella* spp. ^a^, *P. aeruginosa*^a^, *Cryptosporidium* and *Giardia* (oo)cysts ^c^, *Aeromonas* spp. ^e^	River Ruhr and barrier lakes, Germany	NR	Concentrations typically lower and less variable compared to the FIB. No association with precipitation.	[49]
*C. perfringens* ^e^	*L. monocytogenes*^b^, *Salmonella* spp. ^b^, *E. coli* O157:H7 ^b^, *Campylobacter* spp. ^b^, *Cryptosporidium* and *Giardia* (oo)cysts ^c^	South Nation River basin, Canada	Positive, but weak relationships with pathogens.	Correlations with FIB were also weak but positive.	[63]
Somatic coliphage ^e^	Human adenoviruses ^b^	Various rivers and lakes in France, Germany, Italy, Netherlands, Poland, United Kingdom	Concentrations of somatic coliphage correlated with frequency of adenovirus detection.	FIB showed better correlation with adenovirus than somatic coliphage. Somatic coliphage concentrations comparable to *E. coli* concentrations.	[64]
**Marine, and brackish waters**					
*C. perfringens* ^e^	*V. vulnificus*^b^, *S. aureus*^e^, enterovirus ^d^, norovirus ^d^, hepatitis A virus ^d^, *Cryptosporidium* and *Giardia* (oo)cysts ^c^	Virginia Key Beach, Florida, USA	NR	Higher concentrations in high tide samples as opposed to low tide. Not correlated with FIB.	[77]
*C. perfringens*^e^, F+ coliphage ^e^	*V. vulnificus*^b^, *S. aureus*^e^, enterovirus ^d^, norovirus ^d^, hepatitis A virus ^d^, *Cryptosporidium* and *Giardia* (oo)cysts ^c^	Coastal Beaches, Miami Dade County, Florida, USA	NR	High levels of *C. perfringens* also signaled high levels of all FIB.	[76]
*C. perfringens* ^a^	*Cryptosporidium* and *Giardia* (oo)cysts ^c^	Coastal beaches, Venezuela	No significant correlation.	Detection of *C. perfringens* coincided with human-associated MST markers.	[88]
F+ coliphage ^e^	*L. pneumophila*^b^, *S. aureus*^b^, MRSA ^b^, adenovirus ^b^, enterovirus ^d^, Hepatitis A ^d^, Norovirus ^d^	Malibu beach, California USA	F+ coliphage had strong association with MRSA and *S. aureus* presence.	F+ coliphage had strong association with GI illness.	[79]
*C. perfringens*^e^ and F+ coliphage ^e^	*Salmonella* spp. ^b^, *Campylobacter* spp. ^a^, *Cryptosporidium* and *Giardia* (oo)cysts ^c^, adenoviruses ^a^, enteroviruses ^a^	Docklands, South Yarra and Abbotsford estuaries, Melbourne Australia	NR	Positive correlation between the presence of *C. perfringens* and F+ coliphage.	[89]
Somatic ^e^ and F+ coliphage ^e^	Adenoviruses ^a^	Southern California coastal waters, USA	Presence of adenovirus was significantly correlated with F-specific coliphage.	No correlation between two coliphage types.	[90]
Somatic ^e^ and F+ coliphage ^e^	Adenoviruses ^b^, enteroviruses ^b^, hepatitis A ^b^	Rivers and creeks in California, USA	No significant correlation.	Somatic coliphages detected more frequently than F+. Somatic coliphage were not correlated with total coliforms, but F+ coliphage were positively correlated with total/fecal coliforms but not enterococci.	[91]
F+ coliphage ^a^	Enterovirus ^b^, adenoviruses ^b^	Newport Bay, California, USA	No significant correlation.	Peak concentrations of FIB and F+ coliphage associated with winter storms.	[92]
*C. perfringens*^e^ and coliphage ^e^	*Cryptosporidium* and *Giardia* (oo)cysts ^c^, enteroviruses ^a^	Sarasota Bay, Florida, USA	NR	Alternative indicators for co-detected in samples positive for enteric pathogens. Coliphage levels were significantly influenced by salinity and turbidity.	[81]
Somatic ^e^ and F+ coliphage ^e^, phages infecting *Bacteroides thetaiotaomicron* GA17 ^e^	Enteroviruses ^e^	Coastal Waters, Portugal	NR	Enteroviruses were co-detected with FIB. Genogroup I and II F-specific RNA more common in samples the others. Densities of somatic coliphage were higher than FIB densities and did not correlate with them.	[82]
Somatic ^e^ and F+ coliphage ^e^	Enterovirus^a^, *Cryptosporidium* and *Giardia* (oo)cysts ^c^	St. Lucie Estuary, Florida, USA	No significant correlation.	Somatic coliphage concentrations higher than F+ coliphage. Somatic coliphage correlated with the total coliform concentrations.	[94]
*C. perfringens*^e^ and F+ coliphage ^e^	*Salmonella* spp. ^a^, *Campylobacter* spp. ^a^, *S. aureus* ^e^, *V. vulnificus* ^e^, *V. parahaemolyticus* ^e^	Hawaii streams, USA	*C. perfringens* was marginally associated with *Campylobacter* spp. and *V. parahaemolyticus*.	Concentrations of *C. perfringens* and F+ coliphage comparable.	[100]
*C. perfringens*^e^, F+ coliphage ^e^	Enteroviruses ^b^	Florida Keys, Florida, USA	NR	Enteroviruses co-detected with *C. perfringens* and coliphage.	[87]
Somatic coliphage ^e^	Human Adenovirus ^b^	Coastal beaches in Cyprus, Italy, Portugal, Spain and United Kingdom	No significant correlation.	Somatic coliphage concentrations lower than FIB.	[64]

^1^ Data reporting: most probable number (MPN) ^a^, Presence/absence ^b^, total (oo)cysts ^c^, gene copies ^d^, colony forming units or plaque forming units (CFU/PFU) ^e^, Integrated cell culture (ICC)/MPN (RT)PCR ^f^. ^2^ NR (not reported).

**Table 4:** Relationships between MST markers and various pathogens in in freshwater and marine/brackish waters.

MST marker ^1^	Pathogens ^1^	Location	Relationship ^2^	Comments	Reference
**Freshwater**					
*esp*^a^, LTIIa ^a^, STII ^a^	Pathogenic *E. coli* spp. ^a^, *Cryptosporidium* and *Giardia* (oo)cysts ^b^	Various streams in Pennsylvania, USA	No significant correlation.	All MST markers detected more frequently in samples exceeding recreational water quality guidelines.	[56]
*esp* ^a^	Pathogenic *E. coli* spp. ^a^	Various rivers in Georgia, Kansas, Michigan, North Carolina, New Jersey, Ohio, South Dakota, Tennessee, Texas and Virginia, USA	No significant correlation.	*esp* was present in nine samples that met exceeding recreational water quality guidelines.	[57]
HPyV ^a^, *nifH* ^a^, HF183 ^a^	*Campylobacter* spp. ^c^	Various ponds, rivers and creeks in Florida, USA	No significant correlation.	*Campylobacter* and MST markers co-detected at only one site. HPyV and *nif*H were not detected during the study.	[70]
*gyrB*^c^, *g-Bfra*^c^	Human adenovirus ^c^, human enterovirus ^c^, Norovirus GI and GII ^c^, porcine sapovirus ^c^	Delaware Lake, Madison Lake and East Fork Lake, USA	No significant correlation.	*g-Bfra* detected more frequently and at higher concentrations than *gyrB*. *gyrB* and *g-Bfra* frequently correlated.	[72]
*esp ^a^*, HPyV ^a^, HF183 ^a^	*Cryptosporidium* and *Giardia* (oo)cysts ^b^, infectious enteroviruses ^d^, *Salmonella* spp. ^d^	Lake Carroll, Tampa, FL	No significant correlation.	Higher concentrations of indicators and more frequent pathogen detection following rain events. *esp*, but not HPyV or HF183 correlated with FIB (*E. coli*, enterococci, fecal coliforms).	[48]
Bac32F ^a^, CF128 ^a^, CF193 ^a^, HF134 ^a^, HF183 ^a^, PF163 ^a^	*E. coli* O157:H7 ^a^, *Salmonella* spp. ^a^, *Campylobacter* spp. ^a^	Little Bow and Oldman Rivers, Canada	Positive relationship between detection of Bac32F and all pathogens. Positive relationship between CF128/193 and *E. coli* O157:H7 and *Salmonella* spp. Positive relationship between HF183/134 and *Campylobacter* spp..	Bac32F detected most frequently, followed by CF128/193, PF163 and HF183/134 Pathogens were detected infrequently with *Campylobacter* spp. most commonly detected. Water impacted by agricultural operations.	[106]
HPyV ^c^, HF183 ^c^, AllBac ^c^	*S. aureus*^c^, *Salmonella* spp. ^c^, noroviruses ^c^	Prickett Creek, USA	NR	*Salmonella* spp. were most frequently detected pathogen.	[51]
*esp* ^a^	Infectious enteric viruses ^a^	Lake Michigan, USA	NR	Precipitation and turbidity positively correlated with viruses.	[105]
**Marine and brackish waters**					
HPyV ^a^, *esp* ^a^	*V. vulnificus*^c^, *S. aureus*^c^, enterovirus ^c^, norovirus ^c^, hepatitis A ^c^ virus, *Cryptosporidium* and *Giardia* (oo)cysts ^b^	Virginia Key Beach, Florida, USA	NR	HPyV, *V. vulnificus, Giardia* spp. were co-detected with all FIB and alternative indicators. When FIB levels exceeded regulatory standards, HPyVs and pathogens also detected.	[77]
HPyV ^a^, *esp* ^a^, *Bacteroides thetaiotaomicron* ^a^, BacHum-UCD ^a^ and DogBac ^a^	*V. vulnificus*^c^, *S. aureus*^c^, enterovirus ^c^, norovirus ^c^, hepatitis A ^c^ virus, *Cryptosporidium* and *Giardia* (oo)cysts ^b^	Coastal Beaches, Miami Dade County, Florida, USA	NR	During rain event, DogBac was co-detected with *Cryptospordium, Giardia*, enteroviruses and *V. vulnificus* along with enterococci.	[76]
HF183 ^c^, *C. coccoides* ^c^	*Cryptosporidium* and *Giardia* (oo)cysts ^b^	Coastal beaches, Venezuela	NR	The levels of (oo) cysts varied with the extent of sewage pollution and bather density. HF183 and *C. coccoides* correlated with *C. perfringens*.	[88]
GenBac3 ^c^, HF183 ^a,c^, BacHum-UCD ^c^, *B. dorei* ^a,c^, HumM2 ^c^, HF134 ^a^, HumM19 ^a^, *B. stericoris* ^a^, *B. uniformis* ^c^, *nifH* ^a,c^, *esp* ^c^, HPyV ^a,c^, Gull *Bacteroides* ^a^, C*. marimammalium* ^c^, BacCow-UCD ^c^, BacCan-UCD ^c^	*L. pneumophila*^a^, *S. aureus*^a^, MRSA ^a^, pathogenic *E*. coli ^a^, adenovirus ^a^, enterovirus ^c^, Hepatitis A ^c^, Norovirus ^a^	Malibu beach, California USA	NR	Human-associated MST markers were only predictive of illness at the site known to be impacted by human sewage from faulty infrastructure.	[79]
HPyV ^a^, *nifH* ^a^, HF183 ^a^	*Campylobacter* spp. ^c^	Quietwater Beach, Florida, USA	No significant correlation.	In some instances, MST markers were co-detected with *Campylobacter* especially following rain events.	[70]
*esp*^a^, HPyV ^a^	*Salmonella* spp. ^a^, enteric viruses ^d^	Ben T. Davis and Bahia beaches, Florida, USA	NR	Coxsackie virus B4 and HPyVs were co-detected following a major sewage spill. Fecal coliform concentrations correlated with the *esp* marker HPyV did not correlate with FIB.	[80]
HPyV ^c^, HF183 ^c^, *nifH* ^a^	Adenovirus ^a^	Hillsborough River and St. Johns River, Florida, USA	No significant correlation with HPyV, NR for HF183, *nifH*.	Adenovirus co-detected with HF183 and *nifH*.	[98]
GenBac3 ^a^, HPyV^c^, HF183 ^a^, *nifH* ^a^	Adenovirus ^a^	Hillsborough River, St, Johns River, Ben T. Davis beach, Florida, USA	No significant relationship with HPyV, other MST markers NR	All FIB concentrations exceeded regulatory standards HF183 and *nifH* detected in 80% of samples, whereas adenoviruses were detected in 60% of the samples.	[99]
HPyV ^c^, HF183 ^a^, *nifH* ^a^	Adenovirus ^a^	Avalon and Doheny Beaches, California, USA	At Doheny Beach HPyV and HF183 presence correlated with adenovirus.	Adenovirus not detected at Avalon Beach, impacted by non-point source(s).	[93]
PMMoV ^c^, HF183 ^c^, BacHum-UCD ^c^, *esp* ^a^, *nifH* ^c^	Adenovirus ^a^, Norovirus, *Cryptosporidium* and *Giardia* (oo)cysts ^b^, *C. jejuni* ^a^, *Salmonella* spp. ^a^, *S. aureus* ^a^, *E. coli* 0157-H7 ^a^	Coastal beaches, Florida, USA	NR	PMMoV co-occurred with FIB, other MST markers and pathogens. Seawater samples taken near sewage outfalls.	[83]
BacHum-UCD ^c^, HF183 ^c^, DogBac ^c^, *C. marimammalium* ^c^	*S. aureus* ^e^	Coastal beaches, Miami, Florida, USA	NR	Co-occurrence with *S. aureus* detection. No correlation found with MST markers and skin illness.	[86]

^1^ Data reporting: Presence/absence ^a^, total (oo)cysts ^b^, gene copies ^c^, most probable number (MPN) ^d^, colony forming units (CFU) ^e^; ^2^ NR (not reported).

**Table 5:** Frequency (%) of detection of microorganisms over all eligible studies (those that included data on individual observations). Detection frequency is expressed per study and for cumulative samples across all studies. Studies with least one sample positive for the organism were scored positive in the “per study” column.

Organism	Detection Frequency per Study (%) and *n* ^2^	Detection Frequency per Sample ^2^	Detection Frequency per Study (%) and *n* ^2^	Detection Frequency per Sample ^2^
	Freshwater		Brackish/Marine	
**FIB**				
Total coliforms (MPN)	100% (4)	100% (275)	N/A	N/A
Total coliforms (CFU)	100% (3)	97.2% (1988)	100% (5)	99.7% (317)
Fecal coliforms (MPN)	100% (4)	100% (147)	N/A	N/A
Fecal coliforms (CFU)	100% (6)	96.7% (1726)	100% (11)	98.3% (524)
*E. coli* (MPN)	100% (8)	97.7% (1846)	100% (5)	100% (55)
*E. coli* (CFU)	100% (10)	90% (2530)	100% (8)	94.1% (406)
*E. coli* (Q)	100% (5)	89.6% (221)	N/A	N/A
Enterococci (MPN)	100% (2)	81.7% (301)	100% (5)	61.7% (162)
Enterococci (CFU)	100% (13)	96.6% (2584)	100% (13)	97.6% (705)
Enterococci (Q)	100% (2)	100% (302)	100% (3)	100% (34)
**Alternative indicators**				
*C. perfringens* (CFU)	100% (4)	83.2% (1843)	100% (5)	61.6% (73)
*C. perfringens* (Q)	100% (2)	73.2% (56)	N/A	N/A
Somatic coliphage (PFU)	100% (4)	85.5% (394)	100% (1)	100% (20)
F+ coliphage (PFU)	100% (2)	93.2% (73)	100% (7)	34.4% (90)
F- coliphage (PFU)	N/A	N/A	100% (3)	28% (25)
*B. fragilis* phage (PFU)	N/A	N/A	100% (1)	16.7% (12)
*B. thetaiotaomicron* phage (PFU)	N/A	N/A	100% (1)	30% (20)
**MST markers**				
GenBac3 (Q)	100% (1)	75% (8)	N/A	N/A
HF183 (E)	N/A	N/A	100% (3)	31.8% (255)
HF183 (Q)	N/A	N/A	100% (4)	25.5% (105)
BacHum-UCD (Q)	N/A	N/A	100% (1)	95.45% (22)
HPyV (E)	100% (1)	0% (18)	100% (4)	51.08% (204)
HPyV (Q)	100% (1)	100% 98)	100% (2)	12.2% (255)
*C. coccoides* Human (Q)	N/A	N/A	100% (1)	69.2% (13)
*B. thetaiotamicron* (E)	N/A	N/A	100% (1)	26.7% (15)
*nifH* (E)	100% (1)	0% (18)	100% (2)	2.8% (255)
*nifH* (Q)	N/A	N/A	100% (1)	100% (7)
*gyrB* (Q)	100% (1)	50.8% (65)	N/A	N/A
*g-Bfra*	100% (1)	92.3% (65)	N/A	N/A
*esp* (E)	100% (3)	6.2% (649)	80% (5)	19.12% (204)
LTII (E)	100% (1)	7.4% (217)	N/A	N/A
STII (E)	100% (1)	4.6% (217)	N/A	N/A
DogBac (Q)	N/A	N/A	100% (1)	86.7% (15)
**Bacterial pathogens**				
*E. coli* O157:H7 (MPN)	100% (1)	0.6% (823)	N/A	N/A
*E. coli* O157:H7 (E)	100% (1)	13.4% (67)	0% (1)	0% (7)
Pathogenic *E. coli* (*eae*) (E)	100% (4)	53.7% (350)	N/A	N/A
Pathogenic *E. coli* (*eae*) (Q)	100% (1)	31.3% (32)	N/A	N/A
Pathogenic *E. coli* (*stx1*) (E)	100% (4)	7.9% (302)	N/A	N/A
Pathogenic *E. coli* (*stx2*) (E)	100% (3)	29.7% (350)	N/A	N/A
*Salmonella* spp. (MPN)	100% (6)	14% (1076)	33% (3)	8.7% (196)
*Salmonella* spp. (E)	N/A	N/A	100% (1)	28.6% (7)
*Salmonella* spp. (Q)	100% (4)	27.3% (1188)	N/A	N/A
*S. aureus* (MPN)	100% (1)	44.6% (112)	100% (2)	70.4% (27)
*S. aureus* (E)	N/A	N/A	100% (1)	57.1% (7)
*S. aureus* (Q)	100% (1)	37.5% (8)	N/A	N/A
*Campylobacter* spp. (MPN)	100% (3)	24.7% (1009)	100% (1)	30.9% (55)
*Campylobacter* spp. (E)	N/A	N/A	100% (1)	14.3% (7)
*Campylobacter* spp. (Q)	100% (3)	81.3% (80)	100% (1)	18.2% (11)
*Pseudomonas* spp. (MPN)	100% (2)	80.6% (191)	N/A	N/A
*P. aeruginosa* (Q)	100% (2)	39.6% (53)	N/A	N/A
*Shigella* spp. (E)	100% (1)	6.3% (48)	N/A	N/A
*Shigella* spp. (Q)	100% (2)	14.5% (1148)	N/A	N/A
*Legionella* spp. (E)	100% (2)	41.9% (217)	N/A	N/A
*Legionella* spp. (Q)	100% (1)	20% (30)	N/A	N/A
*Listeria* spp. (MPN)	100% (1)	18.7% (395)	N/A	N/A
*V. cholerae* (Q)	100% (2)	52.5% (1148)	N/A	N/A
*V. vulnificus* (Q)	N/A	N/A	100% (2)	44.4% (27)
*Aeromonas* spp. (Q)	100% (3)	100% (248)	N/A	N/A
*M. avium* (Q)	100% (2)	34.4% (64)	N/A	N/A
**Viral pathogens**				
Infectious enterovirus (MPN)	100% (3)	18.4% (158)	100% (3)	0% (27)
Enterovirus (E)	100% (3)	14.4% (222)	100% (3)	37.8% (45)
Enterovirus (Q)	100% (4)	29.1% (103)	33% (3)	14.8% (27)
Infectious reovirus (MPN)	100% (1)	21.9% (32)	N/A	N/A
Reovirus (Q)	100% (1)	100% (8)	N/A	N/A
Human adenovirus (MPN)	N/A	N/A	100% (1)	0% (27)
Human adenovirus (E)	100% (4)	42.2% (1118)	100% (6)	16.5% (309)
Human adenovirus (Q)	100% (5)	40.7% (214)	100% (1)	33.3% (12)
Astrovirus (E)	100% (1)	15.4% (52)	N/A	N/A
Astrovirus (Q)	100% (2)	17.3% (133)	N/A	N/A
Norovirus (E)	100% (3)	29.5% (112)	100% (1)	27.3% (22)
Norovirus (Q)	100% (1)	100% (8)	50% (2)	26.3% (19)
Norovirus GI (Q)	50% (4)	7.5% (213)	N/A	N/A
Norovirus GII (Q)	66.7% (3)	16.2% (198)	N/A	N/A
Rotavirus (E)	100% (3)	18.4% (141)	N/A	N/A
Rotavirus (Q)	66.7% (3)	33.8% (142)	N/A	N/A
Hepatitis A (E)	N/A	N/A	100% (1)	80% (10)
Hepatitis A (Q)	100% (1)	1.5% (68)	0% (1)	0% (12)
Bovine adenovirus (Q)	100% (1)	56.7% (30)	N/A	N/A
**Protozoan pathogens**				
*Acanthamoeba* spp. (E)	100% (1)	24.6% (126)	N/A	N/A
*Naegleria* spp. (E)	100% (1)	13.5% (126)	N/A	N/A
*Cryptosporidium* spp. (M)	88.9% (9)	36.5% (1456)	60% (5)	23.2% (112)
*Cryptosporidium* spp. (E)	N/A	N/A	0% (1)	0% (12)
*Cryptosporidium* spp. (Q)	N/A	N/A	100% (1)	26.7% (15)
*Giardia* spp. (M)	100% (8)	63.7% (1426)	80% (5)	22.3% (112)
*Giardia* spp. (E)	N/A	N/A	100% (2)	16.7% (12)
*Giardia* spp. (Q)	N/A	N/A	100% (1)	33.3% (15)
**Fungal pathogens**				
*C. albicans* (MPN)	N/A	N/A	100% (1)	45.4% (152)

^1^ MPN, most probable number; CFU, colony forming units; PFU, plaque forming units; E, end-point PCR; Q, qPCR; M, microscopy; ^2^ N/A, not available.

**Table 6:** Concentrations of various indicators and pathogens from select studies in marine/brackish and freshwaters.

Organism ^1^	Range or Average per Study (log_10_ per 100 mL) ^2,3^	Marine References ^4^	Freshwater References ^4^
**FIB**			
Total coliforms (c)	2.67–3.89	N/A	[37,75]
Fecal coliforms (c)	0.93–5.39	[76,77,80,87,94]	[39,57,65,125]
*E. coli* (c)	0.59–3.51	[76,77,80,82,89,94,96,100]	[36,37,44,46,50,51,54,55,56,57,65,75,125]
*E. coli* (q)	1.75–3.97	N/A	[41,51,55,68]
Enterococci (c)	ND–3.62	[70,76,77,80,82,83,84,85,87,88,89,94,96,100,101]	[37,39,44,47,50,51,55,56,57,65,70]
Enterococci (q)	0.63–3.21	[76,77,83]	[51,55]
**Alternative indicators**			
*C. perfringens* (c)	0.20–2.33	[76,77,87,88,89,100]	[54]
*C. perfringens* (q)	0.35	N/A	[41]
Somatic coliphage (c)	0.61–3.29	[82,90,94]	[39,102,103,126]
F-specific coliphage (c)	ND–2.76	[76,82,89,90,94,100]	[47,54,102,103]
**MST markers**			
AllBac(q)	0.93	[83]	[51]
PMMoV (q)	ND	[83]	N/A
HF183 (q)	ND–0.69	N/A	[51]
HBBac (q)	0.72	[83]	N/A
HPyV (q)	2.36	N/A	[51]
*esp* (q)	0.06	[76]	N/A
*nifH* (q)	ND	[83]	N/A
DogBac (q)	2.50	[76]	N/A
**Bacterial pathogens**			
*Campylobacter* spp. (c)	1.05	N/A	[54]
*Campylobacter* spp. (q)	0.74–2.29	N/A	[46,70]
EHEC (q)	0.13	N/A	[65]
*Salmonella* spp. (c)	ND–2.36	[80,84,89,100]	N/A
*Salmonella* spp. (q)	0.06–4.08	N/A	[51,65,68]
*S. aureus* (c)	0.70–0.77	[76,77]	N/A
*S. aureus* (q)	0.78	N/A	[51]
*Aeromonas* spp. (q)	5.09	N/A	[68]
Stx1 (q)	0.03	N/A	[57]
Stx2 (q)	0.03	N/A	[57]
*Mycobacterium* spp. (q)	3.41	N/A	[68]
*Pseudomonas* spp. (c)	0.37–3.72	N/A	N/A
*Legionella* spp (q)	ND	N/A	[46]
*V. vulnificus* (c)	1.91–2.11	[84,100]	N/A
*V. parahaemolyticus* (q)	1.01	[85]	N/A
**Viral pathogens**			
Enterovirus (c)	ND–0.04	[80,82,87,89,94]	[39,47,102]
Enterovirus (q)	ND–0.56	[76,77]	[39]
Adenovirus (q)	0.08–1.02	N/A	[41,46,103]
Astrovirus (q)	1.48	N/A	[103]
Norovirus (q)	ND–1.40	[77,94]	[51,102]
Norovirus GI (q)	ND–1.02	N/A	[50,103]
Norovirus GII (q)	1.58	N/A	[103]
Rotavirus (q)	1.41–1.53	N/A	[102,103]
Reovirus (c)	0.18–0.31	N/A	[47,102]
Hepatitis A (q)	ND	[77]	N/A
**Protozoan pathogens**			
*E. bieneusi* (m)	0.12	[101]	N/A
*Giardia* spp.(m)	ND–1.93	[76,83,88,89,94,96,127]	[36,41,47,54,55,56,65,75]
*Cryptosporidium* spp. (m)	ND–0.73	[76,77,83,88,89,94,96,127]	[36,41,47,54,55,56,65,75]

^1^ c, culture; q, qPCR; m, microscopy. ^2^ Range is provided when more than one study measured a given parameter, while average per study is provided when a single study measured a given parameter. Units include: CFU, MPN, PFU, gene copies or total (oo)cysts. ^3^ ND, not detected; ^4^ N/A, not available.
